# Assessment of Genetic Heritability in Rice Breeding Lines Based on Morphological Traits and Caryopsis Ultrastructure

**DOI:** 10.1038/s41598-020-63976-8

**Published:** 2020-05-08

**Authors:** Subhas Chandra Roy, Pankaj Shil

**Affiliations:** Plant Genetics & Molecular Breeding Laboratory, Department of Botany, University of North Bengal, PO-NBU, Siliguri, 734013 WB India

**Keywords:** Plant sciences, Plant breeding

## Abstract

Rice (*Oryza sativa* L) is a most important staple food crop of the world because more than half of the World’s population is dependent on it for their livelihood. Global rice production must be doubled by 2050 to cope up with the situation of population growth. Narrow genetic base in the released varieties has made the improvement in plateaus. Widening the genetic base is necessary to overcome the yield barrier. Hybridization and pre-breeding has been carried out to broaden the genetic base. Heritability and genetic advances were measured in the F5 lines (Tulaipanji × IR64), F3 lines (Tulaipanji × IR64 × PB1460), and F3 lines (Badshabhog × Swarna sub1). Some of the breeding lines were showing promising field performance with high yield potentiality. Wide crosses were performed to widen the genetic base between (Ranjit × *O. rufipogon*) and (Badshabhog × *O. rufipogon*) and the heritability pattern of the morphological characteristics in the progeny lines was evaluated. Nutritional quality of the rice grain is totally dependent on the morphology and histological characteristics of the caryopsis which are genetically determined. Caryopses ultrastructural analyses were carried out in seventeen different rice breeding lines through SEM. SEM analysis showed distinguishing ultrastructure in respect to pericarp, testa, aleurone layer, protein bodies and starchy endosperm in the breeding lines with distinctive inheritance pattern. This study provides information about the cross compatibility of the wide hybridization and heritability measures of the morphological traits which may supplement the breeding program to break the yield plateaus.

## Introduction

Rice (*Oryza sativa* L.) is most important and staple food crop because more than half of the world’s population (>3.5 billion) depends on it for their livelihood^1–3^. It is cultivated in more than 100 countries worldwide^4^. The genus *Oryza* (including both cultivated and wild rice species) can grow in diverse climatic conditions and locations from the wettest areas to the driest deserts in the world ranging 53° north to 40° south latitude, from sea level to an altitude of 3000 m above sea level^5^. Due to this wide range of growing habitat under diversified climatic conditions, the genus *Oryza* shows a wide range of genetic and phenotypic diversity^6^ with a maximum diversity existing in wild rice species because they have not undergone rigorous human selection^7–11^. The production of double the amount of rice by 2050 is urgently needed to feed more than 9 billion people in this world^12,13^. The production rate in the released varieties has plateaued due to narrow genetic base in the parental lines used in breeding programs^14,15^. Genetic bottleneck during domestication also causes erosion of the genetic diversity in the well adapted cultivars which leads to yield stagnation^15^. Yield plateaus can be surmounted through genetic gain by combining the yield related genes/QTLs from different genetic resources of rice germplasm both from local landraces and crop wild relatives. Wild species are the reservoir of genetic diversity and wide adaptability. Therefore, germplasm diversity is considered as the mainstay for crop improvement. Breeder can introgress these genetic gain related genes/QTLs using knowledge of molecular breeding techniques such as marker assisted selection^16–19^. The rice germplasm is a rich reservoir of valuable genes which has accumulated over a period of time and is yet to be exploited fully by plant breeders for varietal improvement^20^. Highly recombined populations such as MAGIC (Multi-parent Advanced Intercross) has been developed to maximize the genotypic diversity in order to increase genetic enhancement^21–23^. Genetic variability, heritability and genetic advance and QTLs mapping in rice has been studied^24–28^ to analyse the yield and yield components for crop improvement.

Rice fruit is a caryopsis in which the single seed is fused with the wall of the ripened ovary (pericarp) forming a seed-like grain. The rice grain (rough rice) consists of an edible part, the rice caryopsis, which is protected by a covering known as hull (husk). Husk contains the larger lemma and smaller palea jacketing the rice caryopsis^29–31^. Rice caryopsis consists of pericarp, testa, aleurone, embryo and starchy endosperm. Starchy endosperm is accounting for more than 90% of the caryopsis and contains parenchyma cells filled with storage components representing a major source of food for humanity^32^. Caryopsis size and number of the aleurone layers determine the quantities of important phytochemicals and minerals (zinc and iron) in the grains^33^. One of the components of yield increase is the grain weight. Hence, an understanding about the ultrastructure and histology of the caryopsis and its relationship to grain weight is needed^34^. Structure and developmental stages of the caryopsis has been studied through histochemical staining and scanning electron microscopy (SEM) to understand their developmental characterstics^11,32,35,36^. Histologically, rice caryopsis is composed of cuticular layer (CL), Aleurone layer (AL), and endosperm layer (EL), in centripetal order^37,38^. Both the layer (CL and AL) together is termed as bran layer and are generally removed during milling and polishing. Consumption of unpolished rice (Brown rice, BR) may conserve about 43–54 million tonnes rice every year^39^. Brown rice has greater human health benefits as well as increase in grain yield due to the presence of bran layer^40^. These layers (CL, AL) plays vital role in cooking behaviour of the rice grains too^41^.

There is no report about the improvement of rice cultivars Tulaipanji and Badshabhog through breeding and also no report regarding the pre-breeding between Ranjit × *O. rufipogon*; Badshabhog × *O. rufipogon* for broadening the genetic base. Therefore, the objectives of this study was to assess the genetic variability, heritability and genetic advance (GA) of yield and yield associated traits in some promising breeding lines (Tulaipanji × IR64); (Tulaipanji × IR64 × PB1460); (Badshabhog × Swarna Sub1); and pre-breeding lines (Ranjit × *O. rufipogon*) and (Badshabhog × *O. rufipogon*) to assist the future breeding programs for yield improvement by widening the genetic base and genetic recombination. Morpho-histological (ultrastructure) characteristics of the rice caryopsis have also been investigated *in situ* conditions before and after cooking (retrograded stage) through scanning electron microscope (SEM) to trace the inheritance patterns of these traits in the breeding lines.

## Results

### Breeding lines developed

**Figure 1:**
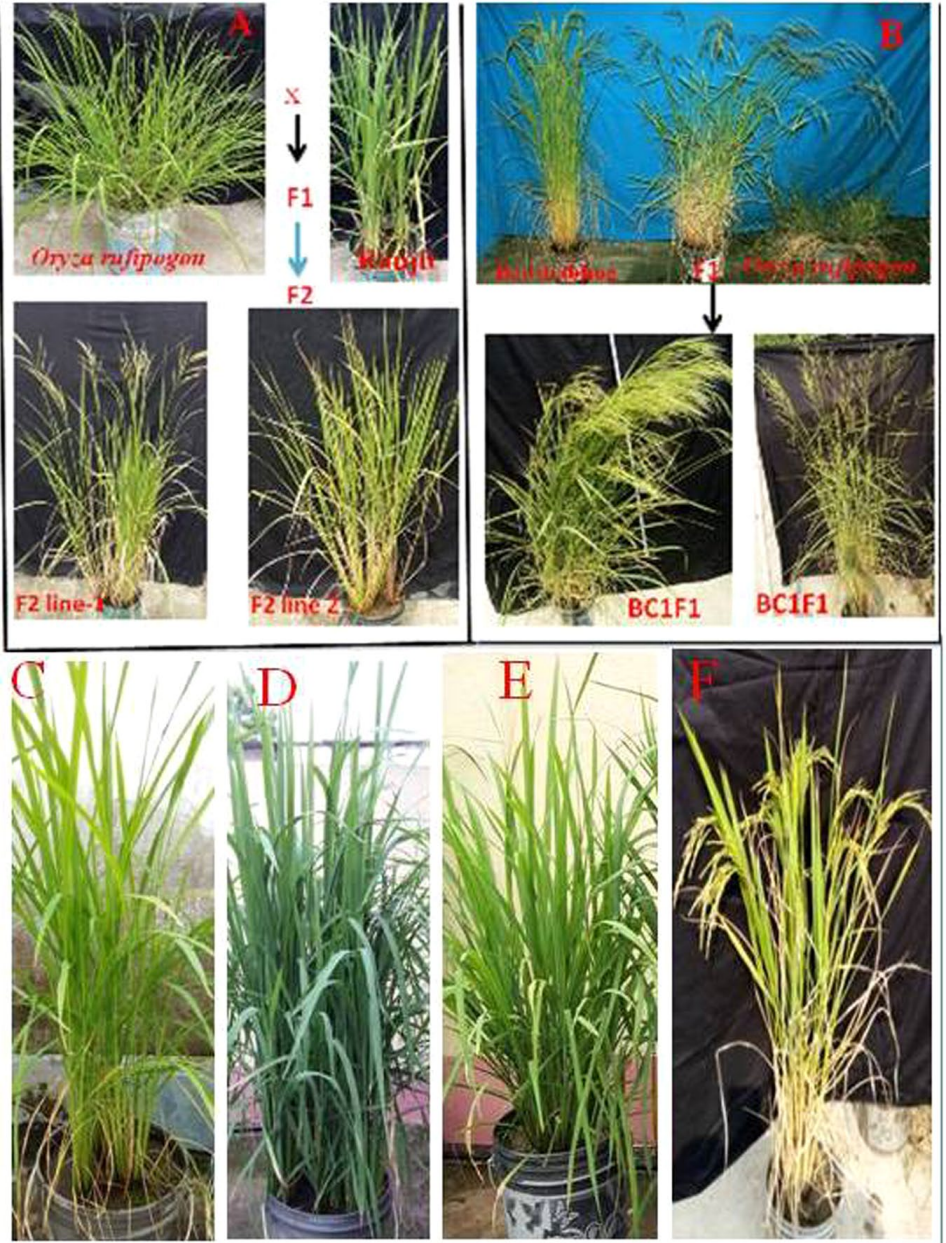
Hybridization between *Oryza sativa* and *Oryza rufipogon* in Pre-breeding program. (**A**) Wide cross Ranjit × *O. rufipogon* (**B**) Badshabhog × *O. rufipogon* also BC1F1, (**C**) Tulaipanji, (**D**) PB-1460, (**E**) IR64, and (**F**) Swarna Sub1.

**Figure 2:**
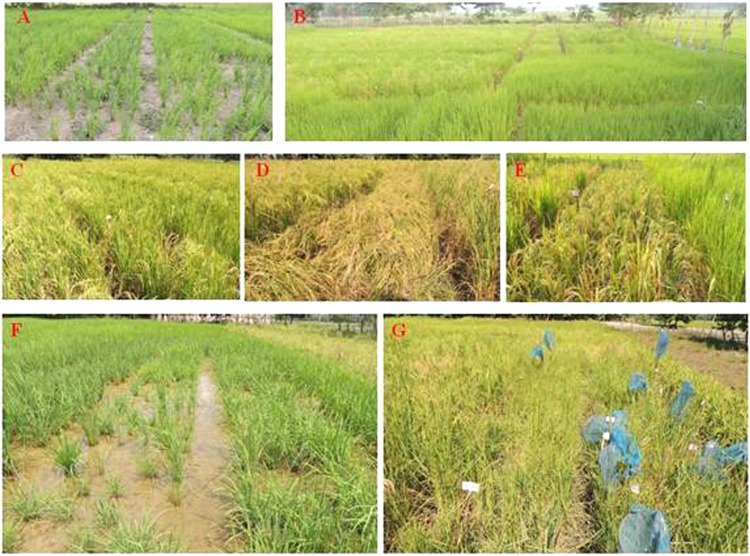
Performance of the breeding lines in the field conditions (kharif 2018). (**a**) Progeny F5 lines (Tulaipanji × IR64) at early vegetative stage, (**b**). Progeny F5 lines (Tulaipanji × IR64), F3 (Tulaipanji × IR64 × PB1460), F2 (Badshabhog × Swarna Sub1), F2 (Ranjit × *O. rufipogon*), and F2 (Badshabhog × *O. rufipogon*) along with parental lines in flowering stage, (**c**). Progeny F5 lines in ripening stage, (**d**). Progeny F5 with parental line Tulaipanji maturity stage (complete lodging), (**e**). Breeding lines F3(Tulaipanji × IR64 × PB1460) ripening stage, (**f**). Pre-breeding lines F2 (Ranjit × *O. rufipogon*), and F2 (Badshabhog × *O. rufipogon*) at vegetative stage, (**g**). Shattered mature seeds of pre-breeding F2 progeny lines (Ranjit × *O. rufipogon* and Badshabhog × *O. rufipogon*) were collected in synthetic mesh covering.

Three different cross combinations were developed in the intra-specific hybridization program. These crosses were (Tulaipanji × IR64) having progeny generation lines in F_5:6_, (Tulaipanji × IR64 × PB1460) having progeny lines in F_2:3_; and (Badshabhog × Swarna sub1) having progeny lines in F_2:3_ stage. Two wide cross combinations were established in the pre-breeding program for the introgression of agronomically important traits (gene/QTLs) into the cultivars. These prebreeding lines were at F_2:3_ populations between (Ranjit × *O. rufipogon*) and between (Badshabhog × *O. rufipogon*) and maintained for genetic evaluation purpose (Fig. 1). All the breeding lines were evaluated at the field level (Fig. 2) based on DUS test protocol of PPV&FR Acts 2001, Govt. of India.

### Estimation of variability parameters

The ANOVA revealed that there is highly significant differences existed among the breeding lines considering the agro-morphological traits (Supplementary Tables S1–S5). Traits are comprising of plant height, flag leaf length, flag leaf width, panicle length, grain per panicle, grain length, grain breadth, 1000 grain weight, maturity time (days), active tillering number, awn length and aroma. It was observed in the present study that there were inherent genetic differences among the breeding lines (genotypes) in respect to the morphological traits considered during the analysis. Existence of high variability in the breeding lines will increase the probability of producing desirable recombinants in successive generations. Genetic variability was analysed in all the breeding lines (Tables 1–5 and Supplementary Figs. S1–S5) and correlation among the paired traits of the five different breeding populations are summarized (Supplementary Tables S6–S10). Variability parameters of different biometrical traits of the breeding lines are depicted in Table 1 for progeny lines F_5:6_ (Tulaipanji and IR64), in Table 2 for progeny lines of F_2:3_ (Tulaipanji × IR64 × PB1460), in Table 3 for cross (Badshabhog × Swarna Sub1) of F_2:3_ progeny lines. Results of the interspecific hybridization (pre-breeding) were summarized in Table 4 for F_2:3_ progeny lines (Ranjit × *O. rufipogon*) and in Table 5 for F_2:3_ progeny lines (Badshabhog × *O. rufipogon*).

**Table 1:** Descriptive statistics variability parameters of different agro-morphological traits for population F_5:6_ (Tulaipanji × IR64) was analyzed. Plant height (PH), flag leaf length (FLL), flag leaf width (FLW), panicle length (PnL), grain per panicle(Gr/Pn), grain length (GL), grain breadth (GB), 1000 grain weight (Gr/Wt), active tillering (Till), heading date (HD), and maturity time (MT).

Traits	Mean ± SE	Range	GV	PV	GCV%	PCV%	H (%)	Skewness	Kurtosis	GA	GAM
PH	133.98 ± 0.28	118.00–145.00	1.85	2.63	1.01	1.21	70	−0.107	0.129	2.35	1.75
FLL	27.10 ± 0.11	18.00–36.00	2.16	2.58	5.44	5.94	83.74	−0.177	1.075	2.77	10.27
FLW	13.86 ± 0.11	12.50–15.50	0.33	0.34	4.17	4.27	95.34	0.226	0.188	1.15	8.40
Pnl	29.33 ± 0.19	20.00–37.00	11.98	12.12	11.80	11.86	98	−0.187	−0.677	7.09	24.20
Gr/Pn	209.72 ± 2.81	90.00–330.00	2680.76	2686.91	24.68	24.71	99.77	−0.236	0.018	106.93	50.98
GL	8.91 ± 0.06	7.00–10.95	1.8058	1.8062	15.0819	15.0836	99.97	0.301	−1.288	2.76	31.02
GB	2.54 ± 0.01	1.85–2.95	0.1046	0.1054	12.73	12.78	99.16	−1.106	−0.086	0.67	26.46
GrWt	25.03 ± 0.33	13.5–31.30	42.03	42.33	25.90	25.99	99.29	−0.759	−1.249	13.31	53.19
Till	19.90 ± 0.33	18.00–25.00	4.27	4.37	10.45	10.57	97.71	0.529	−1.233	4.21	21.31
HD	85.82 ± 0.33	80.00–95.00	44.16	44.16	7.74	7.74	100	0.242	−1.632	13.70	15.97
MT	116.66 ± 0.22	110.00–125.00	89.70	89.70	8.06	8.06	100	0.056	−1.388	19.53	16.62

**Table 2:** Descriptive statistics variability parameters of different agro-morphological traits for population F_2:3_ (Tulaipanji × IR64 × PB1460) was analyzed. Plant height (PH), flag leaf length (FLL), flag leaf width (FLW), panicle length (PnL), grain per panicle(Gr/Pn), grain length (GL), grain breadth (GB), 1000 grain weight (Gr/Wt), active tillering (Till), heading date (HD), and maturity time (MT).

Traits	Mean ± SE	Range	GV	PV	GCV%	PCV%	H (%)	Skewness	Kurtosis	GA	GAM
PH	117.50 ± 0.70	85.00–133.00	62.94	99.85	6.75	8.50	63.03	−1.01	0.57	12.99	11.05
FLL	23.68 ± 0.32	12.00–32.00	3.40	12.92	7.78	15.17	26.31	−0.40	1.01	1.95	8.24
FLW	12.74 ± 0.06	10.00–14.50	0.04	0.36	1.56	4.70	11.14	−0.08	1.64	0.13	1.08
PnL	28.34 ± 0.35	18.00–38.00	17.81	24.85	14.89	17.58	71.68	0.20	−0.91	7.37	26.00
Gr/Pn	179.20 ± 2.47	110.00–240.00	874.58	1224.51	16.50	19.52	71.42	−0.21	−1.02	51.56	28.77
GL	10.65 ± 0.02	9.50–11.80	0.08	0.10	2.63	2.99	77.45	−0.08	1.05	0.51	4.78
GB	2.32 ± 0.01	1.76–2.65	0.01	0.05	3.60	9.60	14.00	−0.63	−0.76	0.06	2.75
GrWt	26.22 ± 0.20	20.00–30.00	8.54	8.74	11.11	11.27	97.72	−0.27	−1.25	5.96	22.73
Till	14.01 ± 0.15	10.00–15.00	0.74	1.96	6.10	9.99	37.75	−1.33	0.67	1.09	7.78
HD	95.90 ± 0.54	90.00–100.00	24.47	24.47	5.15	5.15	100.00	−0.37	−1.91	10.20	10.64
MT	127.95 ± 0.27	125.00–130.00	6.12	6.12	1.93	1.93	100.00	−0.37	−1.91	5.10	3.99

**Table 3:** Descriptive statistics variability parameters of different agro-morphological traits for population F_2:3_ (Badshabhog × Swarna Sub1) was analyzed. Plant height (PH), flag leaf length (FLL), flag leaf width (FLW), panicle length (PnL), grain per panicle(Gr/Pn), grain length (GL), grain breadth (GB), 1000 grain weight (Gr/Wt), active tillering (Till), heading date (HD), and maturity time (MT).

Traits	Mean ± SE	Range	GV	PV	GCV%	PCV%	H (%)	Skewness	Kurtosis	GA	GAM
PH	146.04 ± 1.18	127.00–168.00	119.30	131.12	7.40	7.80	90.00	−0.03	−1.05	21.49	14.71
FLL	25.53 ± 0.74	13.00–38.00	27.94	51.48	17.76	24.11	53.27	−0.50	−0.83	8.03	26.99
FLW	13.20 ± 0.07	11.00–15.00	0.28	0.56	4.06	5.70	50.91	−0.33	0.45	0.78	5.97
PnL	30.95 ± 0.39	25.00–45.00	11.82	15.03	11.10	12.52	78.00	1.10	1.42	6.28	20.29
Gr/Pn	269.05 ± 5.41	180.00–375.00	1104.86	2752.07	12.35	19.40	40.00	0.34	−0.62	43.44	16.14
GL	8.48 ± 0.04	7.00–9.70	0.39	0.43	7.38	7.76	90.00	−0.16	−0.48	1.22	14.38
GB	2.47 ± 0.01	2.20–2.95	0.02	0.04	6.27	7.78	64.00	0.97	0.19	0.26	10.40
GrWt	19.50 ± 0.27	13.00–26.00	12.90	13.13	18.41	18.58	98.00	−0.15	−1.07	7.34	37.64
Till	21.41 ± 0.54	16.00–29.00	19.36	27.98	20.55	24.70	69.00	0.49	−1.40	7.55	35.26
HD	85.53 ± 1.10	60.00–90.00	115.30	115.30	12.00	12.00	100.00	−2.00	2.06	22.15	25.89
MT	115.53 ± 1.10	90.00–120.00	115.30	115.30	12.00	12.00	100.00	−2.00	2.06	22.15	19.17

**Table 4:** Descriptive statistics variability parameters of different agro-morphological traits for population F_2:3_ (Ranjit × *O. rufipogon*) was analyzed. Plant height (PH), flag leaf length (FLL), flag leaf width (FLW), panicle length (PnL), grain per panicle(Gr/Pn), grain length (GL), grain breadth (GB), 1000 grain weight (Gr/Wt), active tillering (Till), heading date (HD), and maturity time (MT).

Traits	Mean ± SE	Range	GV	PV	GCV%	PCV%	H (%)	Skewness	Kurtosis	GA	GAM
PH	157.11 ± 2.35	110.00–231.00	782.24	861.05	17.80	18.67	90.00	0.26	−0.08	54.99	35.00
FLL	33.40 ± 0.75	15.00–45.00	48.30	57.08	20.81	22.62	84.61	−0.64	−0.70	13.18	39.48
FLW	13.70 ± 0.16	10.00–16.00	0.52	2.68	5.26	11.94	19.40	−0.46	−0.65	0.66	4.78
PnL	25.65 ± 0.34	16.00–36.00	13.50	18.84	14.32	16.92	71.00	0.45	−0.56	6.41	24.99
Gr/Pn	120.62 ± 3.45	25.00–225.00	1525.53	1850.48	32.38	35.66	82.00	0.16	−0.01	73.16	60.65
GL	8.12 ± 0.03	7.40–8.90	0.15	0.21	4.70	5.50	71.00	0.28	−1.40	0.66	8.12
GB	2.70 ± 0.01	2.25–3.00	0.01	0.03	3.09	6.08	25.92	−0.50	−0.37	0.09	2.06
GrWt	16.60 ± 0.10	13.50–18.50	1.60	2.08	7.61	8.68	77.00	−0.63	−0.79	2.29	13.79
Till	24.31 ± 0.57	15.00–33.00	26.87	45.33	21.27	27.62	59.27	−0.09	−1.03	8.23	33.78
HD	91.87 ± 0.24	90.00–95.00	5.92	5.92	2.64	2.64	100.00	0.53	−1.76	5.01	5.46
MT	130.93 ± 0.20	130.00–135	3.85	3.85	1.40	1.40	100.00	1.63	0.66	4.04	3.09

**Table 5:** Descriptive statistics variability parameters of different agro-morphological traits for population F_2:3_ (Badshabhog × *O. rufipogon*) was analyzed. Plant height (PH), flag leaf length (FLL), flag leaf width (FLW), panicle length (PnL), grain per panicle(Gr/Pn), grain length (GL), grain breadth (GB), 1000 grain weight (Gr/Wt), active tillering (Till), heading date (HD), and maturity time (MT).

Traits	Mean ± SE	Range	GV	PV	GCV%	PCV%	H (%)	Skewness	Kurtosis	GA	GAM
PH	146.04 ± 1.18	127.00–168.00	119.30	131.12	7.40	7.80	90.00	−0.03	−1.05	21.49	14.71
FLL	25.53 ± 0.74	13.00–38.00	27.94	51.48	17.76	24.11	53.27	−0.50	−0.83	8.03	26.99
FLW	13.20 ± 0.07	11.00–15.00	0.28	0.56	4.06	5.70	50.91	−0.33	0.45	0.78	5.97
PnL	30.95 ± 0.39	25.00–45.00	11.82	15.03	11.10	12.52	78.00	1.10	1.42	6.28	20.29
Gr/Pn	269.05 ± 5.41	180.00–375.00	1104.86	2752.07	12.35	19.40	40.00	0.34	−0.62	43.44	16.14
GL	8.48 ± 0.04	7.00–9.70	0.39	0.43	7.38	7.76	90.00	−0.16	−0.48	1.22	14.38
GB	2.47 ± 0.01	2.20–2.95	0.02	0.04	6.27	7.78	64.00	0.97	0.19	0.26	10.40
GrWt	19.50 ± 0.27	13.00–26.00	12.90	13.13	18.41	18.58	98.00	−0.15	−1.07	7.34	37.64
Till	21.41 ± 0.54	16.00–29.00	19.36	27.98	20.55	24.70	69.00	0.49	−1.40	7.55	35.26
HD	85.53 ± 1.10	60.00–90.00	115.30	115.30	12.00	12.00	100.00	−2.00	2.06	22.15	25.89
MT	115.53 ± 1.10	90.00–120.00	115.30	115.30	12.00	12.00	100.00	−2.00	2.06	22.15	19.17

### Estimation of heritability and genetic advance in rice breeding lines

The estimation of GCV, PCV, heritability, genetic advance and genetic advance as percent of mean (GAM) were analyzed in all the breeding lines (Tables 1–5). The PCV were higher that of the corresponding GCV value indicated the little influence of the environment in each trait (interacted with the environment to some extent). Agro-morphological traits were studied (Table 1) in the present investigation for cross (Tulaipanji × IR64) considering plant height (cm), panicle length (cm), grain per panicle, grain length (mm), grain breadth (mm), and 1000 grain weight (g). Phenotypic coefficient of variation (PCV %) was high (24.68%) in compare to genotypic coefficient of variation (GCV %) for the trait grain number per panicle (24.65%), for grain weight PCV and GCV value was 25.99 and 25.90 respectively. Heritability value was highest in the trait grain length (99.97%) followed by 99.77%, 98%, and 99.29% in grain number per panicle, plant height, and grain weight respectively. Genetic advance (GA) was quite high 106.93 for the trait grain number per panicle with genetic advance as mean of percentage (GAM) 50.98.

High heritability was observed in the breeding lines of the cross (Tulaipanji × IR64 × PB1460) in the F_2:3_ mapping populations (Table 2). The trait grain breadth showed very low heritability (14.00%) in this population. High amount of genetic advance was observed in the traits pertaining to grain number per panicle (51.56) with high value of genetic advance of percentage of mean (28.77). In case of the cross (Badshabhog × Swarna Sub1), some of the traits were showing high heritability such as plant height (90%), panicle length (78%), grain length (90%), and grain weight (98%) (Table 3). Highest heritability was detected for the trait 1000 grain weight (98%) with highest GAM value 37.64. Grain per panicle showed low heritability (40%). Pre-breeding interspecific hybridization lines also showed tremendous genetic variability in the F_2:3_ progeny lines while analyzed the eleven morphological traits (Tables 4 and 5). Low heritability was observed in the trait of grain breadth only 25.92% in the cross Ranjit × *O. rufipogon* (Table 4). Which is indicating that the trait is not heritable and that cannot be used in further selection process. High heritability and high genetic advance was found in the trait grain per panicle in this wide cross 82% and 73.16 respectively. Genetic advance as percentage of mean value was also high (60.65) in this trait, which indicating that the trait is governed by additive gene action (Table 4). In the second wide hybridization (Badshabhog × *O. rufipogon*), heritability calculation was high for the traits plant height, panicle length, grain length, grain breadth and 1000 grain weight but less in the trait for grain number per panicle (Table 5).

### Histological ultrastructural architecture of rice caryopsis (*in situ*)

**Figure 3:**
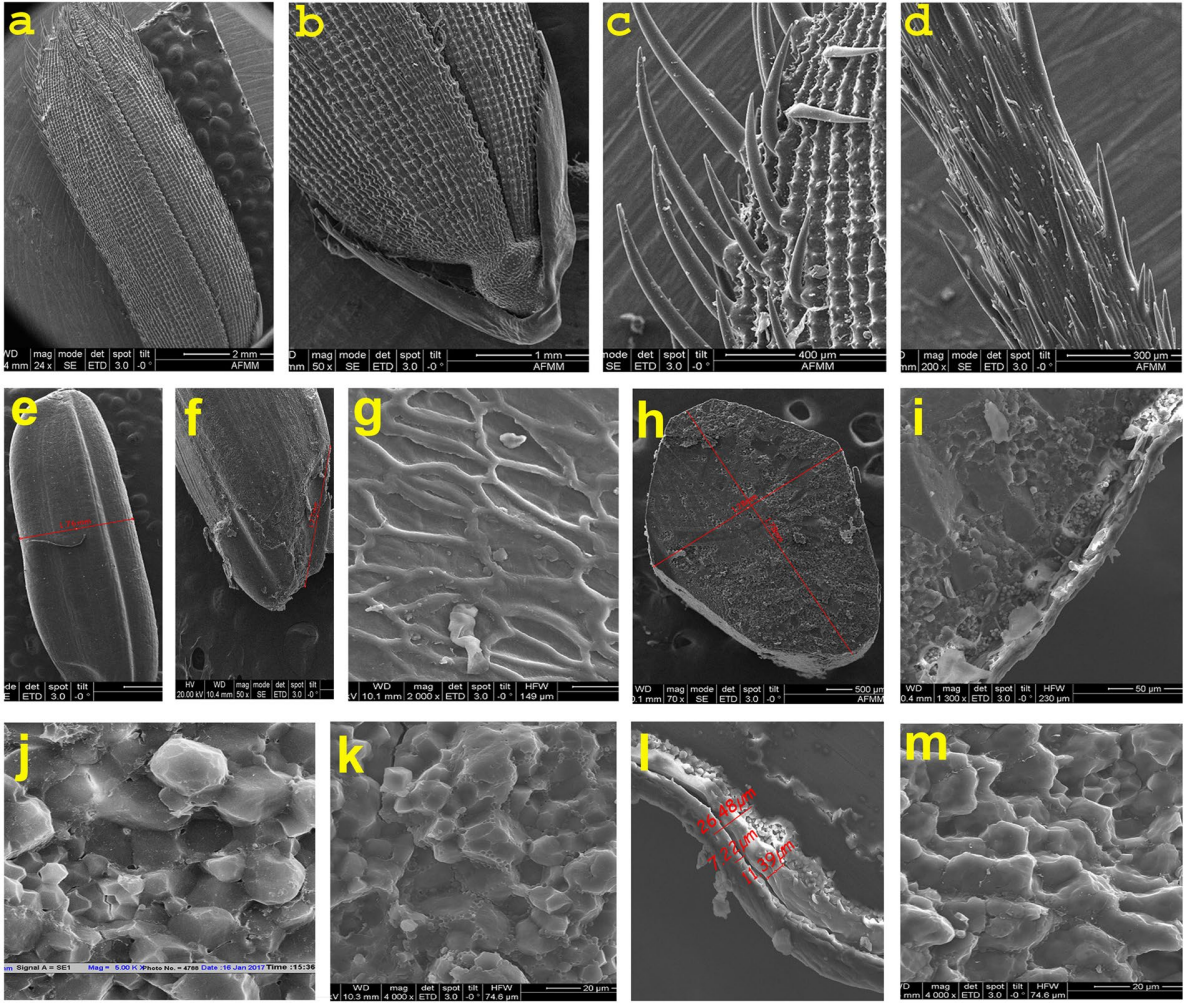
Scanning electron microscopic (SEM) images (as one representative example) of wild rice (*Oryza rufipogon*), uncooked, (**a**–**k**); cooked and retrograded (***l-m***): (**a**) Morphology of whole seed grain, (**b**). enlarged glumes and upper surface of the seed, (**c**). magnified view of hairs on the seed surface morphology, (**d**). awn surface, (**e**). morphology of whole kernel upper surface (caryopsis), (**f**)*.* embryo location on the caryopsis, (**g**). reticulate network like microstructural architectures of caryopsis upper surface, (**h**)*.* transverse cross section of whole caryopsis showing histological characteristics, (**i**). histological ultrastructural architecture of pericarp, testa, aleurone layer and starchy endosperm with starch granules (polyhedral to spherical in shape), microspore pinholes and protein bodies at the peripheral side of the starch granule, (**j,k**)*.* Magnified view of ultrastructural morphology of starch granules, compound starch granule (CSG) and protein bodies (PB), (**l**). Retrograded caryopsis with deformed pericarp, testa and aleurone layer after cooking, and (**m**). endosperm layer with CSG is in degraded conditions in retrograded caryopsis.

**Figure 4:**
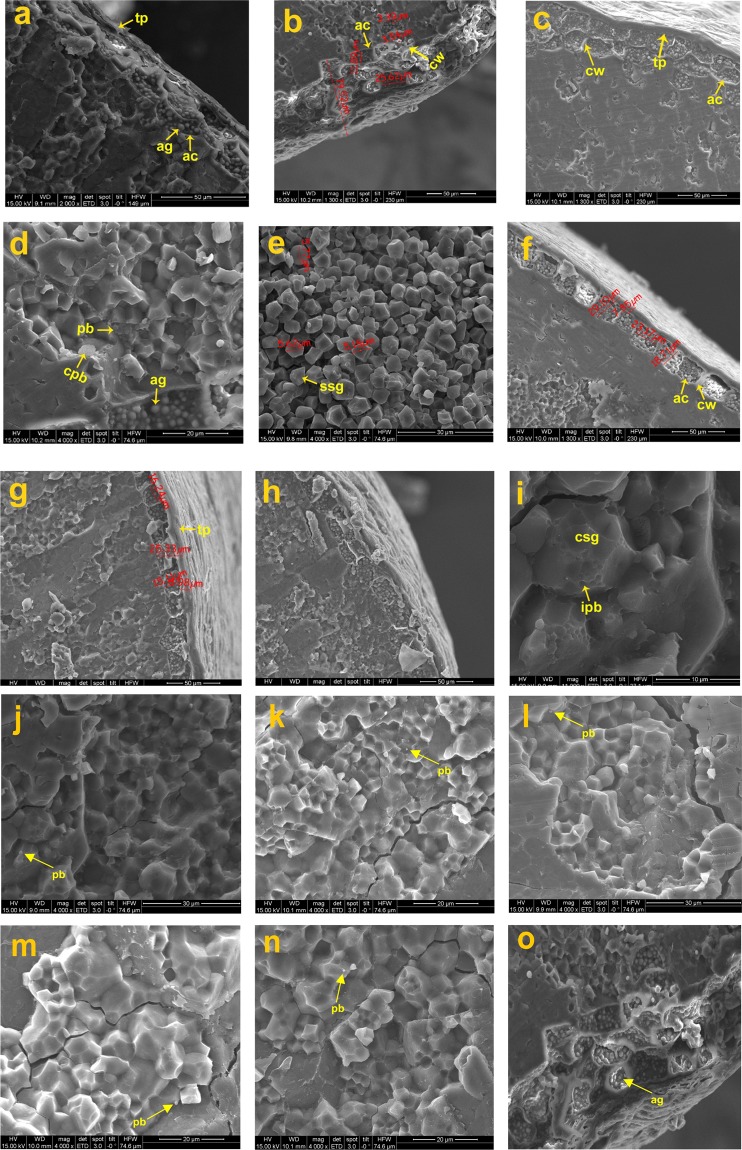
Scanning electron microscopic (SEM) images of the parental and breeding lines: (**a**). IR64, (**b**). F5AN, (**c**)*.* F5AL, (**d**). F5AN with PB, (**e**). F5AL retrograded with single starch granule, (**f**)*.* F5Blk, (**g**)*.* F5MdG, (**h**)*.* Tulaipanji bran layer, and (**i**). Tulaipanji with protein impression, (**j**)*.* Tulaipanji with PB (**k**). IR64 with PB, (**l**). F5AL with PB, (**m**). F5Blk with PB*,* (**n**). F5MdG with PB, and (**o**). F5AN with enlarged view of aleurone layer cell with aleurone grain (ag).

**Figure 5:**
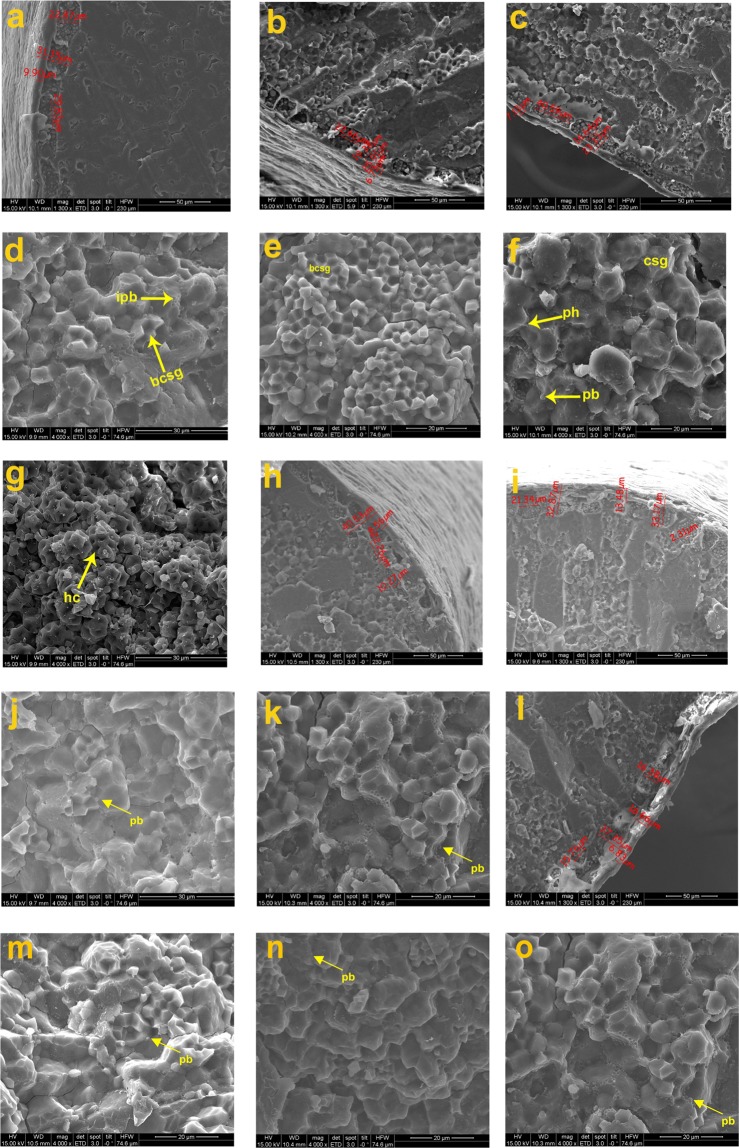
Scanning electron microscopic (SEM) images of the parental and breeding lines: (**a**). PB1460, (**b**). F2i, (**c**). F2ii, (**d**). F2i with Protein body, (**e**). F2i with CSG, (**f**). F2ii with PB, (**g**). PB1460 with honey-comb like structure in retrograded condition, (**h**). Ranjit, (**i**). RRF2 (Ranjit × *O. rufipogon*), (**j**). RRF2 with protein body, (**k**). Wild rice (*O. rufipogon*) with PB, and (**l**). Wild rice (*O. rufipogon*), (**m**). Ranjit with PB, (**n**). PB1460 with PB, and (**o**), *O. rufipogon* with PB.

**Figure 6:**
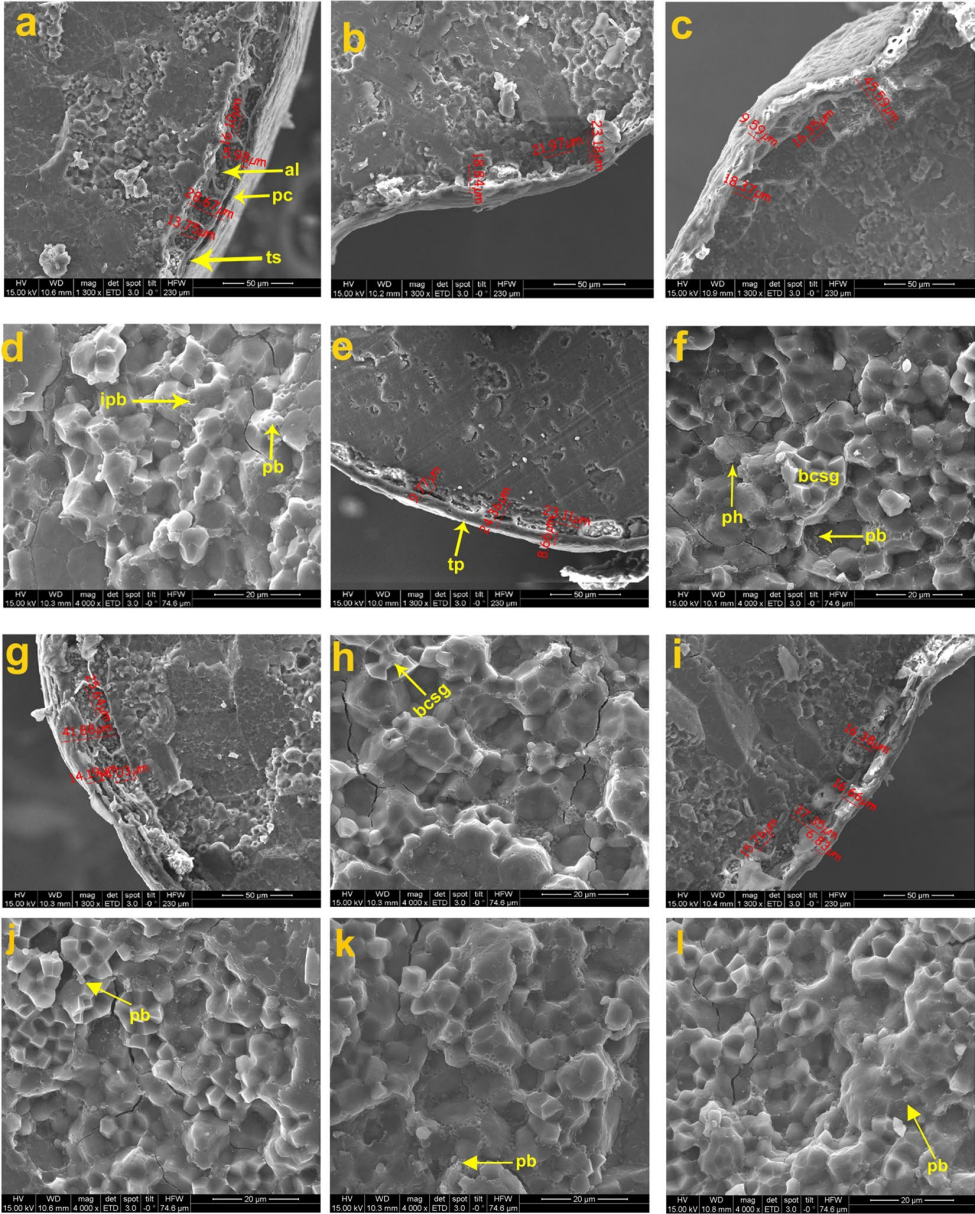
Scanning electron microscopic (SEM) images of the parental and breeding lines: (**a**). Swarna sub1 (SSub1), (**b**). BGSF2A (Badshabhog × Swarna Sub1), (**c**). BGSF2B, (**d**). BGSF2A with PB, (**e**). BDbhog (Badshabhog), (**f**). BDbhog with PB, (**g**). BGRF2 (Badshabhog × *O. rufipogon*), (**h**). BGRF2 with PB, and (**i**). Wild rice (*O. rufipogon*), (**j**). Swarna Sub1 with PB, (**k**). BGSF2B with PB, and (**l**). *O. rufipogon* with PB.

**Figure 7:**
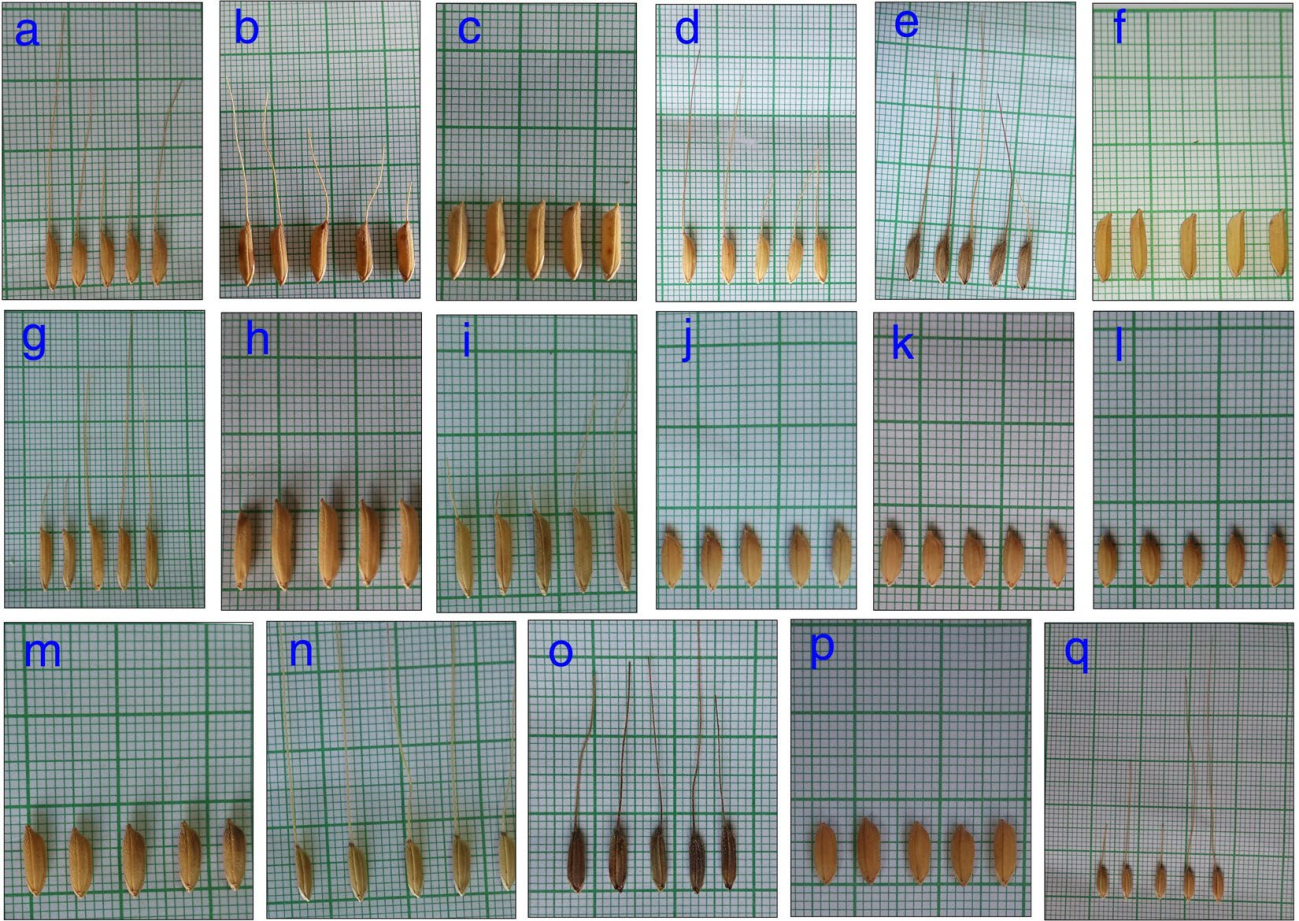
Grain morphology of rice varieties and different breeding lines are summarized. (**A**) Tulaipanji, (**B**). F5AN, (**C**). F5AL, (**D**). F5Blk, (**E**). F5MdG, (**F**). IR64 (**G**). PB1460, (**H**). F2i, (**I**). F2ii, (**J**). BGSF2A, (**K**). BGSF2B, (**L**). Badshabhog, (**M**). Ranjit, (**N**). RRF2, (**O**). *O. rufipogon* (**P**)*.* Swarna Sub1, and (**Q**). BGRF2.

Different ultrastructural layers of the caryopsis were investigated based on SEM captured images at different magnification (30X, 700X, 1300X, 2000X). Histological characteristics of the cooked retrograded caryopsis were studied at *in situ* condition by SEM and morpho-histological changes were detected. Histological ultrastructural architecture of the outer most pericarp layer, then testa (seed coat), aleurone layer and endosperm layer in centripetal manner were clearly observed *in situ* by SEM in different rice varieties and breeding lines before and after cooking (Figs. 3–6). Summary of the comparative histological architectural characteristics observed distinctly in the 17 (seventeen) rice lines caryopsis were represented to trace the inheritance pattern (Table 6). Physicochemical properties such as alkali spreading value (ASV), aroma, gelatinization temperature (GT) and grain morphology were also studied (Table 7, Fig. 7).

**Table 6:** Summary of the caryopses ultrastructural characteristics studied under scanning electron microscope (SEM) of the breeding lines.

Rice varieties	Thickness Pericarp-testa (μm)	Size of the Aleurone layer cell (μm) and area	Bran Thickness (μm)	Size AG (μm)	Size CSG (μm)	Size PB (μm)	Abundance of PB
IR64	10.01	10.07 × 26.53 = 267.15	34.87	0.32–3.93	7.31–12.50	0.43–1.29	**+**
Tulaipanji	8.97	10.05 × 31.62 = 317.78	24.24	0.43–4.30	3.01–11.61	0.86–2.15	**++**
F5AL	7.63	19.81 × 23.92 = 473.85	61.71	0.65–3.93	5.05–12.90	0.43–1.72	**+**
F5AN	7.90	11.89 × 25.62 = 304.62	79.52	0.32–3.54	8.60–12.90	0.43–1.72	**+++**
F5Blk	7.85	18.71 × 23.17 = 433.51	29.10	1.32–3.93	4.30–17.20	0.43–1.29	**+**
F5MdG	8.98	15.11 × 16.24 = 245.38	25.33	0.65–3.94	5.16–12.90	0.86–1.92	**+**
*O. rufipogon*	6.83	9.25 × 35.25 = 326.06	37.35	0.70–4.23	5.64–16.50	1.40–4.30	**+++**
Ranjit	8.56	10.27 × 36.68 = 376.70	40.53	2.62–5.24	6.45–23.65	0.65–1.96	**+**
RRF2	13.48	19.65 × 30.13 = 592.05	33.17	1.31–3.93	3.87–19.35	0.86–1.29	**++**
PB1460	7.07	21.02 × 21.15 = 444.57	30.12	0.70–4.93	5.17–18.80	0.47–1.41	**+**
F2i	6.73	19.38 ×22.66 = 439.15	25.91	0.65–6.55	4.30–12.04	0.43–0.86	**+++**
F2ii	7.13	16.64 × 40.59 = 675.41	23.33	1.31–5.24	5.16–22.36	0.43–2.15	**+++**
Badshabhog	8.66	9.77 ×23.11 = 225.78	24.36	1.38–4.20	4.70–19.00	1.50–4.50	**+++**
BGRF2	14.19	12.28 × 25.64 = 314.85	41.88	0.70–5.65	5.60–18.80	0.47–1.41	**++**
SSub1	5.98	13.75 × 16.10 = 221.37	28.67	0.70–2.82	4.70–16.46	0.47–1.41	**+**
BGSF2A	4.27	14.10 × 28.39 = 400.29	45.02	0.70–2.82	4.70–18.80	1.41–3.52	**+++**
BGSF2B	9.59	10.00 × 18.37 = 183.70	45.59	2.82–7.00	3.76–11.75	1.41–3.52	**+**

Note: F5 lines (Tulaipanji × IR64), F5AL (awnless); F5AN (Awn), F5Blk (black husk), F5MdG (medium grain); RRF2 (Ranjit × *O. rufipogon*); F2i [F2 line of (Tulaipanji × IR64) × PB1460]; F2ii [F2 line of (Tulaipanji × IR64) × PB1460]; BGRF2 (F2 of Badshabhog × *O. rufipogon*); SSub1 (Swarna Sub1); BGSF2A- BGSF2B (Badshabhog × SSub1).

**Table 7:** Summary of the grain morphology with physicochemical properties (alkali spreading value, gelatinization temperature, and aroma) in the rice breeding lines.

Rice varieties	Grain length (mm)	Grain breadth (mm)	1000 Grain weight (g)	Aroma	ASV	GT
IR64	9.64	2.47	24.23	0	1	7
Tulaipanji	7.85	1.87	15.12	3	4	3
F5AL	10.23	2.27	28.38	3	4	3
F5AN	10.06	2.40	27.86	3	3	5
F5Blk	8.22	2.33	19.40	2	4	3
F5MdG	8.65	2.37	22.00	1	3	5
*O. rufipogon*	8.52	2.11	17.00	1	4	3
Ranjit	7.84	2.21	19.27	0	1	7
RRF2	7.68	2.15	22.43	0	3	5
PB1460	10.60	1.94	24.60	1	7	1
F2i	10.55	2.14	26.44	2	4	3
F2ii	10.20	1.81	19.33	2	7	1
Badshabhog	6.13	1.98	10.60	2	7	1
BGRF2	6.86	2.19	14.10	2	3	5
SSub1	7.63	2.53	20.97	0	2	7
BGSF2A	7.20	2.58	17.20	0	2	7
BGSF2B	6.90	2.45	16.20	0	2	7

Note: F5 lines (Tulaipanji × IR64), F5AL (awnless); F5AN (Awn), F5Blk (black husk), F5MdG (medium grain); RRF2 (Ranjit × *O. rufipogon*); F2i [F2 line of (Tulaipanji × IR64) × PB1460]; F2ii [F2 line of (Tulaipanji × IR64) × PB1460]; BGRF2 (F2 of Badshabhog × *O. rufipogon*); SSub1 (Swarna Sub1); BGSF2A- BGSF2B (Badshabhog × SSub1).

Whole caryopsis and cross view was studied under SEM to reveal the detailed architecture of the grain under *in situ* conditions (Figs. 3–6). Rice grain varies in weight 10–30 mg depending on the varietal characteristics. Grain weight may distribute in different parts of the caryopsis ultrastructure which are as follows- pericarp (1–2%), seed coat and aleurone (5–6%), embryo (2–3%) and starchy endosperm (89–94%)^53^. Rice caryopsis is consisting of pericarp, testa, aleurone, embryo and starchy endosperm centripetally. Starchy endosperm is accounting for more than 90% of the caryopsis and contains parenchyma cells filled with storage starch components representing a major source of food for humanity^32^. In the present study under SEM, we have analyzed details histological ultrastructure of the caryopsis encompassing pericarp, testa, aleurone layer, and starchy endosperm. As detected by SEM images, pericarp and testa remain fused in most of the cultivars and breeding lines, aleurone and endosperm layer were morphologically distinct tissue systems of rice caryopsis. Mostly, all the rice cultivars, wild rice and breeding lines have shown more or less similar tissue layers in the caryopsis. Closely associated layer of tissue such as pericarp and testa remain fused together, and termed as cuticular layer (CL). The outer most pericarp was thick and dense without any porous structure like pinhole porosity. Underneath to the pericarp layer is a testa that is a seed coat. Immediately beneath the testa was the aleurone layer (AL), a single layer of cuboidal aleurone cells, sometimes multi cell layer (Figs. 3–6). The thick cell walls of the aleurone cell were clearly visible under SEM images (Figs. 3–6). The aleurone cell always contains loosely packed and scattered aleurone grains (AG), lipid bodies and intracellular voids (Figs. 3–6). The innermost endosperm layer (EL) was compact without intracellular voids. The EL was highly organized by polyhedral starch granule (SG) and ellipsoidal/spherical protein bodies (PB) (Figs. 3–6). Starch granules tightly clustered into compound starch granule (CSG). These CSG were surrounded by protein body (PB) if present in the rice varieties. Compound starch granules (CSG) size varies from smallest (3.01 μm in Tulaipanji) to largest in Ranjit (23.65 μm) in the present investigation (Figs. 4i and 5m). Shape and arrangement of the CSG in the endosperm amylopast plays important role in grain quality. Generally starch granules are consisting of amylose (0–30%) and amylopectin (70% or more than 70%). Thin cell wall contains very little amount of cell wall materials of the endosperm layer. Shape of the CSG was polyhedral to spherical with different size (5.64 μm to 16.50 μm) with minimum angularity in wild rice (*O. rufipogon*), surrounded by many numbers of small size protein bodies (PB) (1.40 μm to 4.30 μm) more than that of Tulaipanji rice and also impression of PB in the marginal side (Fig. 3). Starch granule in IR64 is polyhedral in shape with 3.01 μm to 4.30 μm in size. Compound starch granule (CSG) ranges from 7.31 μm to 12.50 μm with high angularity and compact polyhedral structure in IR64 (Fig. 4). In Tulaipanji, CSG is compact polyhedral structure with moderate or minimum angularity (3.01μm to 11.61 μm in size). Deformed aleurone layer was detected in cooked grain and microspore-pinhole like structure also found (Fig. 4). CSG are various shape and size, hexagonal to polyhedral with moderate angularity (5.05 μm to 12.90 μm in size) in F5AL line. PB is moderately present with less impression of PB (0.43 μm to 1.72 μm in diameter) (like IR64) (Fig. 4l). CSG are very loosely arranged and distinctly separated in the cooked retrograded grains (Fig. 4e). It was a unique features found in this line, hexagonal to polyhedral in shape (5.18 μm to 5.77 μm in size). CSG is polyhedral in shape with moderate angularity (8.60 μm to 12.90) in F5AN line and retrograde grain showed features as like as Tulaipanji. Starch granules exhibit less angular, polyhedral CSG, with moderate protein bodies (6.45 μm to 23.65 μm), less impressions of PB in Ranjit (Fig. 5m). The CSG ranges from 3.87 μm to 19.35 μm in wide cross line RRF2 (Ranjit × *O. rufipogon*) (Fig. 5j). Starch granules are polyhedral in shape with moderate to less angularity. Channels were present in the retrograded grain and less deformed CSG with degraded protein granules were detected. CSG was polyhedral in shape with different in size variation with less or no angularity (5.17 μm to 18.80 μm) in PB1460 (Fig. 5n). Retrograded CSG showing hollow depression just like honeycomb structure or rose-flower like arrangement without any PB or impression of PB in this cultivar PB1460 (Fig. 5g).

CSG are hexagonal to polyhedral in shape (4.30 to 12.04 μm) in F2i line (Tulaipanji × IR64 × PB1460) with variation in size and less angularity. The CSG are mostly polyhedral in shape (5.16 μm to 22.36 μm) with less angularity in F2ii line (Tulaipanji × IR64 × PB1460) (Fig. 5). In case of Badshabhog, CSG were in different shape and size from polyhedral to spherical like (4.70 to 19.00 μm) with minimum angularity, which are surrounded by huge number of small PB (1.5 μm to 4.50 μm) (like wild rice) and impression of PB also found in endosperm layer (Fig. 6). CSG were spherical like to polyhedral in shape (5.60 μm to 18.8 μm) with less angularity of different sizes surrounded by huge number of PB (0.47 μm to 1.41 μm) observed in BGRF2 line (Badshabhog × *O. rufipogon*) and impression of PB in the marginal region but less than the *O. rufipogon* (Fig. 6). CSG was polyhedral in shape with sharp angularity (4.70 μm to 16.45 μm) in Swarna Sub1. CSG in case of breeding line BGSF2A (Badshabhog × Swarna sub1) were spherical like in shape (4.70 to 18.8 μm) and surround by large number of PB (1.41 μm to 3.52 μm, *like O. rufipogon*). Impression of PB with moderate pinholes was also observed in this BGSF2A line (Fig. 6). Other line BGSF2B of the same cross showed polyhedral to spherical like CSG (3.76 μm to 11.75 μm) in addition to small numbers of PB (like IR64) along with impression of PB (1.41μm −3.52 μm) and moderate pinholes in the endosperm layer. Protein bodies (PB) are the rich source of proteins, vitamins and other nutritional properties. Abundance of protein bodies were varies from cultivar to cultivar and designated as low (+), medium (++) and high (+++). Highest amount of PB (+++) was detected in the rice lines F5AN, *O. rufipogon*, F2i, F2ii, Badshabhog, and BGSF2A (Table 6).

From Table 6, it has been observed that the thickness of combined pericarp and testa lowest (4.27 μm) in the breeding line BGSF2A (Badshabhog × Swarna sub1), and highest (14.19 μm) in breeding line BGRF2 (Badshabhog × *O. rufipogon*). More than 10 μm thick layer was recorded in the following genotypes- IR64 and RRF2. Aleurone cell layer area was recorded highest in F2ii line (675.41 μm) and lowest in the breeding line BGSF2B (183.70 μm), followed by 592.05 μm in RRF2. Bran thickness highest was observed in breeding line F5AN (79.52 μm), then 62.71 μm in F5AL (Tulaipanji × IR64), minimum thickness was 24.24 μm in Tulaipanji, 24.36 μm in Badshabhog, and 23.33 μm in F2ii line (lowest). Smallest AG was found in IR64 (0.32 μm) (Table 6). Compound starch granules (CSG) size varies from smallest (3.01 μm in Tulaipanji) to largest in Ranjit (23.65 μm). Shape and arrangement of the CSG in the endosperm amylopast plays important role in grain quality. Generally starch granules are consisting of amylose and amylopectin. Abundance of protein bodies can be of three categories, high abundance in six rice lines (BGSF2A, Badshabhog, F2ii, F2i, *O. rufipogon*, F5AN), medium abundance in three rice lines (BGRF2, RRF2, Tulaipanji) and lowest abundance in eight rice lines (BGSF2B, Swarna sub1, PB-1460, Ranjit, F5MdG, F5Blk, F5AL and IR64). Sensory based aroma content was measured in the varieties with high, medium and low range index. Rice varieties such as –Tulaipanji, Badshabhog, PB1460, wild rice are aromatic, but IR64, Ranjit, Swarna sub1 are non aromatic (Table 7). Some of the breeding lines were inherited fragrance alleles from their parental lines and showed aromatic traits such as F5AN, F5AL, F5Blk, F5MdG, received fragrance allele from Tulaipanji, the F2 lines BGRF2 – has received fragrance allele from Badshabhog, breeding line F2i and F2ii received fragrance allele from Tualiapanji and PB1460 (Table 7). Physicochemical properties of the 17 rice lines were summarized (Table 7) along with their grain morphology (Fig. 7). Out of the 17 rice lines, 11 are aromatic, aroma index were varies from 1 to 3 (sensory based), and remaining 6 rice lines were nonaromatic. Swarna sub1, showed low ASV (2) with high GT (7). Badshabhog showed aroma index value 2, ASV result 7 with low GT value 1 (Table 7).

## Discussion

The present study showed that a wide range of variability was observed for the traits under investigation (Supplementary Tables S1–S5). The presence of high heritability and genetic advance in most of the traits indicating that trait can be improved through direct selection. Estimation of PCV was higher than their corresponding GCV, which signifies that environmental interaction influences the expression of traits (Tables 1–5). Differences between the value of GCV and PCV more than 20%, is considered high, 10–20% is considered moderate and less than 10% are considered low^54^. Higher estimates of GCV and PCV giving the information that the traits were under the genetic control, and the information is helpful for identification of traits during selection (Tables 1–5). Also signifies that these traits could be improved through hybridization and selection in rice breeding program for better yield performance^55–57^. Higher magnitude of GCV and PCV for grain number per panicle was estimated in the cross Tulaipanji × IR64 (Table 1), which supports the view of earlier study^56^. High heritability values were observed in all the traits of all the breeding lines (Tables 1–5) and facilitate the selection process. Broad sense heritability is considered as high if shows value >60%, medium >50–60% and as low if the value is <50%^58,59^. The GAM was categorized into low (0–10%), medium (10.1–20%) and high (>20.1%)^49^. The heritability estimates alone cannot furnish complete practical importance unless it is studied with genetic advance. Since heritability do not always indicate genetic gain, therefore heritability estimation coupled with genetic advance is more effective for selection. High genetic advance with high heritability was observed in all the traits considered in the present investigation which indicated that these traits were less influenced by the environmental fluctuations. Instead these traits are governed by additive gene action and can easily be selected through phenotypic selection (Tables 1–5). Traits of medium heritability and low genetic advance mean (GAM) character is totally governed by non-additive gene action. In this situation heterosis breeding could be used to improve such kind of traits. These traits could also be improved by selection^60–62^. Observations suggest the possibility of improving these characters by further selection in segregating generations. These findings are consistent with the previous report of breeding program for plant height and other traits^63,64^. The higher value of PCV in compare to GCV value indicates the influence of environment and this higher magnitude also specify the scope for selection of traits. The GCV was found to be less than the PCV for all the traits under present study, indicating that the recorded variation was not only controlled by genotype but also influenced by environmental effects on the expression of the phenotypes. High heritability along with high genetic advance as percent of mean was obtained for grain per panicle, 1000 grain weight, grain length which has signified the selection of these traits for further improvement in these breeding lines. Therefore, we recommend for the selection of the traits like grain per panicle, 1000 grain weight, and panicle length for further improvement in the present investigation (Tables 1–5). Heritability results showed the high value for all the agronomically important traits studied. High heritability and genetic advance as a percent of mean was recognized for grain number per panicle (99.77% and 50.98 respectively) and 1000 grain weight (99.29% and 53.19), which showed that the presence of additive gene action for traits expression can be improved by simple selection in the cross Tulaipanji × IR64 (Table 1). This finding is consistent to the views of other investigators^63–65^. On the other hand, moderate heritability and low genetic advance as a percent of the mean was registered for grain number in cross Badshabhog × Swarna Sub1 (40%, 16.14 respectively), indicating that the environment interacted to express the traits (Table 3). Hence, direct selection for the trait will be less effective. In some results, low heritability and genetic advance suggests that non-additive gene action plays a major effect. Two promising lines can be selected from the F5 breeding lines (Tulaipanji × IR64) which showed enhanced yield and aroma. Grain length was 10.06–10.23 mm, breadth was 2.27–2.40 mm, 1000 grain weight was 27.86–28.38 g, which was just 27.09%, 34.63% and 87.69% improvement over Tulaipanji in respect to these traits. Maturity time has been reduced to 135 days instead of 145–150 days in Tulaipanji. One progeny line F2i of the triparental cross (Tulaipanji × IR64 × PB1460) had given enhanced yield potentiality and quality. Grain quality was judged using standard physicochemical parameters (ASV, sensory based aroma and GT). These breeding lines can be released after more field trail. Positive significant correlations were observed among the traits under study for the five different breeding lines but in some traits it also showed negative correlation (Supplementary Tables S6–S10). This correlation pattern indicated that agronomically important traits can be improved in the breeding lines if a specific trait is improved.

Investigation was performed by imaging the morpho-histological characteristics of rice caryopsis through scanning electron microscope (SEM) to reveal the histological ultrastructural architecture in the rice caryopsis in different varieties and breeding lines in its original location and form (*in situ*). Comparison was done between the uncooked and cooked (retrograded) rice caryopsis to judge the quality of rice grain in terms of cellular components maintenance before and after cooking. Rice grain starches are two categories- hot water soluble and insoluble. During cooking hot water soluble components leach out from the endosperm layer and may form thin film covering around the caryopsis and giving cooked rice individuality. Endosperm cell wall was so thin that it cannot be resolved clearly under SEM, it was consistent with those of earlier finding^66^. Inheritance pattern of caryopsis histological ultrastructure components were investigated in this present study. It was observed that one of the F5 progeny lines (Tulaipanji × IR64 = F5AN) with awn trait showed better parent value in respect to bran thickness in the caryopsis, which was 79.52 μm whereas in the parental lines Tulaipanji and IR64, these were 24.24 μm and 34.87 μm respectively. Other F5 lines of the same cross showed bran thickness below the parental value (Table 6). In this cross, other line F5AL giving the transgressive segregation pattern in respect to trait bran thickness. Similar pattern of transgressive segregation was observed in the traits considering grain length, grain breadth and 1000 grain weight in this progeny line F5AN (Supplementary Table S1, Fig. 7). 1000 Grain weight was  28.37 g in F5AN line, whereas in parental lines value was 15.12 g and 24.23 g in Tulaipanji and IR64 respectively. Transgressive inheritance of the bran thickness largely contributed to the grain weight ( 28.37 g) and consequently to the yield enhancement of the breeding line F5AN (Tulaipanji × IR64). The F5AN line not only enhanced the yield traits also improved the grain quality due to the increase of the abundance of protein bodies (PB) (Table 6). Same type of inheritance was also marked in F5AL line. The cuticular layer (fused pericarp and testa) were intact after cooking condition but inner endosperm layer had been gelatinized completely (Fig. 4e) and have shown deformed CSG and PB at different level. Histological architecture of AL upon cooking were observed and showed more remarkable changes. The thick cell walls of aleurone cells were exposed probably due to the dissolution of cytoplasmic components of aleurone cells. During cooking some channels were formed in the aleurone cell layer and many aleurone grains (AG) were still morphologically visible after cooking under SEM. Whole structural entity of endosperm cells was totally ruptured into a honeycomb-like matrix and other retrograded forms, which indicates that starch granules and protein bodies were thermally unstable (Fig. 5g). Cell walls of endosperm were completely disrupted upon cooking owing to their low thickness and weak mechanical strength (Figs. 4–6). The present finding was consistent with the earlier report^41^. It was noticed that in the retrograded caryopsis, the CL and AL remained morphologically identifiable in spite of hot water boiling during cooking (Figs. 4–6) in some cultivars and breeding lines.

In case of wide interspecific cross, Ranjit × *O. rufipogon*, transgressive inheritance pattern was observed in trait related to cuticular layer (pericarp-testa), which was 13.48 μm thick in RRF2 breeding line, whereas parental line with 6.83 μm (*O. rufipogon*) and 8.56 μm in Ranjit (Fig. 5, Table 6). This transgressive segregating trait may lead to the yield enhancement by increasing the 1000 grain weight (22.43 g) in RRF2. Parental lines 1000 grain weight was 17.00 g and 19.27 g in wild rice (*O. rufipogon*) and Ranjit respectively (Table 6). In case of triparental crossing (F2i - Tulaipanji × IR64 × PB1460) protein body (PB) has been transferred from the parental lines into the progeny lines. The F2i line showed increased grain weight due to the presence of abundance of protein bodies, which also improved the quality parameters (Fig. 5d, Table 6). In case of Badshabhog × *O. rufipogon* cross, pericarp thickness has been enhanced (14.19 μm) compared to 8.66 μm in Badshabhog and 6.83 μm in wild rice *O. rufipogon* (Fig. 6, Table 6). Bran thickness showed transgressive segregation pattern in the breeding line BGRF2 (41.88 μm) whereas parental line showed thickness 24.36 μm in Badshabhog and 37.35 μm in wild rice. Outer cuticular layer thickness (pericarp-testa) was increased in the breeding line BGSF2B (Badshabhog × Swarna sub1) which showed 9.59 μm in thickness whereas 5.95 μm and 8.66 μm thickness was found in Swarna sub1 and Badshabhog respectively (Fig. 6, Table 6). The trait of bran thickness showed inheritance of better parent value (45.02 μm, 45.59 μm) in both the breeding line compared to parental lines, 28.67 μm in Swarna sub1 and 24.36 μm in Badshabhog. Grain weight in both the progeny lines were increased (17.20 g in BGSF2A, 16.20 g in BGSF2B) compared to parental lines Badshabhog (10.60 g) and Swarna sub1 (17.00 g) (Fig. 6, Table 6).

Rice grain weight is one of the determining agronomic traits of crop yield and which is associated with the structural development of the caryopsis^67^. Our present results of caryopsis ultrastructural features also supporting this finding. Grain weight was increased in the progeny F5 lines (Tulaipanji × IR64) such as F5AL ( 29.37 g) and F5AN ( 28.37 g) compared to parental lines (Supplementary Table S1) Tulaipanji (15.12 g) and IR64 (24.23 g). This increment was corresponding to the bran thickness of the caryopsis of F5AL (61.71μm) and F5AN (79.52 μm), which was more than that of the parental lines (Tulaipanji 24.24 μm and IR64 34.87 μm). Grain weight genetic traits (gene/QTLs) are inherited generation after generation with transgressive segregation properties showing high heritability (99.29%), genetic advance (13.31) and high GAM (53.19) in this F5 lines (Tulaipanji × IR64). The grain weight trait in these lines are not influenced by environmental factors because it showed high heritability with high genetic advance which indicating the additive gene action (Table 1). Caryopsis ultrastructural differences under SEM analysis can be used as marker for yield improvement along with the other DNA based marker to detect the phenotype (grain weight) directly. Rice quality is a combination of traits, consisting of milling, appearance, cooking, tasting and nutrient qualities, which is closely related to structural development of the caryopsis. Caryopsis morphology and histological ultrastructure depends on the genotypes of the rice cultivars which are totally governed by the gene/QTLs. Rice quality is affected not only by genetic factors but also by environment and cultivation system^68^. In the present investigation, SEM based caryopsis ultrastructural characteristics were studied in the parental lines as well as progeny breeding lines. Main chemical components of the rice caryopsis is starch (90% in w/w) and consisting of amylose and amylopectin molecules developed in the amyloplast of the endosperm. Grains with well developed starch granule with angular polyhedral appearance make grain transparent and non-chalky with good milling characteristics^69^. But amyloplast spherical in shape with large spaces between plastid, resulted in chalky grains of poor milling quality. Protein bodies were found in the endosperm layer and vary in quantity. Some of the breeding lines (F5AN, F2i, F2ii, and BGSF2A) contain high amount (+++) of PB in their endosperm which indicating that nutrient quality and medicinal property has been improved by breeding and selection and confirmed through ultrastructural observation under SEM (Figs. 4 and 5, Table 6). Our result was consistent with the finding of other^68^. Protein bodies (PB) if accumulated in higher numbers, leads to a higher nutrient value and medicinal property but taste is poor^68^. The developmental status of amyloplasts and protein bodies determines the quality and quantity of rice grains^70,71^. High or low temperature affects the sequence and pattern of enzyme changes during grain filling, resulting in increased rice chalkiness^32,72^. In a rice breeding program, gelatinization temperature (GT) may be measured by the alkali spreading value (ASV) test based on standard protocol^51^, value ranges from 1 to 7. Rice cultivars with higher GT (value 7) must be cooked a few minutes longer that those with lower values (value 1). Low gelatinization temperature rice tend to start absorbing water and swell at lower temperature during cooking than those with starch of higher gelatinization temperature. Breeding lines F5AL showed ASV-4, aroma index-3 and medium GT value 3, but F5AN showed aroma index 3, ASV-3, and GT value 5. Results of GT value are inversely proportional to the value of ASV (Table 7). Aroma index ranged from 0 to 3. Aroma absent then index is 0, aroma highest means index is 3.

## Conclusion

Two promising lines were selected from the cross Tulaipanji × IR64 and one line from triparental cross (Tulaipanji × IR64 × PB1460) with high yield potentiality. Maturity duration has been reduced to 130 days instead of 150 days in parental line Tulaipanji. These breeding lines were photoinsensitive and may grow during summer season as Boro rice. Farmers can cultivate these lines in both the season kharif as well as summer. These promising lines showed high heritability with additive gene action of the phenotypic traits under study and showed immense scopes to improve the varieties through selection. Selection could be effective for the traits grain number, panicle length under present investigation. High heritability along with high genetic advance as percent of mean was observed for grain number per panicle, 1000 grain weight which signifies the selection of these traits may helpful for further crop improvement. Breeding lines of the cross Badshabhog × Swarna Sub1 was conducted to develop high quality improved Swarna Sub1 by transferring aroma and nutritional quality genes from Badshabhog to fulfil the SDG for nutritional security. Caryopsis ultrastructural features (SEM analysis) can be used as phenotypic marker to observe the inheritance of grain traits directly (pericarp, testa, aleurone layer and starch endosperm). SEM ultrastructural study has provided morpho-anatomical distinguishing features of caryopsis *in situ* condition and can help the breeders to take right decision for the selection of the breeding lines to maintain high heritability, genetic advance to continue the yield potentiality. Wide inter-specific hybridization was done between Ranjit × *O. rufipogon* and Badshabhog × *O. rufipogon*. This pre-breeding was aimed to broaden the gene pool (genetic base) of the rice cultivars (*Oryza sativa*) by introgressing agronomically important unadapted gene/QTLs from their wild relatives (*O. rufipogon*) to develop climate ready crop varieties. This is the most demanding breeding strategy in this climate change scenario. The back crossing was performed to develop chromosome segment substitution lines (CSSL) for the generation of near isogenic lines (NIL) with unique specific trait to be used as mapping population to identify gene/QTLs for the important traits.

## Materials and methods

### Plant material

**Figure 8:**
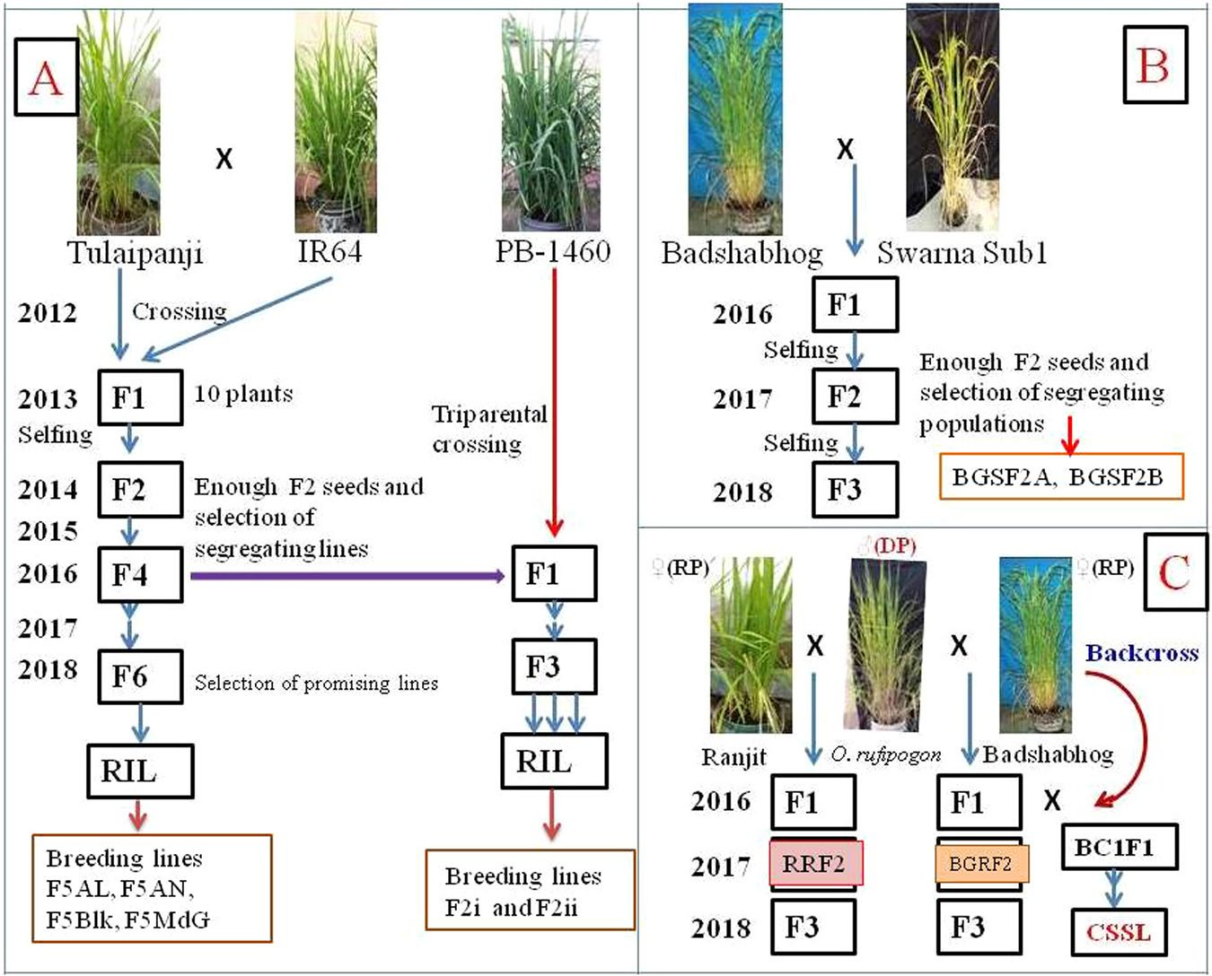
Breeding scheme has been outlined. (**A**) Cross-I between Tulaipanji and IR64, Cross-II between Tulaipanji × IR64 × PB1460, (**B**) Cross-III between Badshabhog × Swarna Sub1, (**C**) Interspecific hybridization, Cross-IV between Ranjit × *O. rufipogon*; Cross-V between Badshabhog × *O. rufipogon* [DP-donor parent, RP-recurrent parent].

Rice [*Oryza sativa* L.) cultivars such as Tulaipanji, Badshabhog, Ranjit, IR64, PB1460, Swarna Sub1 and one wild rice genotype [*Oryza rufipogon* Griff.] were used for the present breeding program to improve the agronomically important traits (Fig. 8). Intraspecific breeding lines were developed between [Tulaipanji × IR64; Tulaipanji × IR64 × PB1460; Badshabhog × Swarna Sub1] and two interspecific breeding lines were also developed [Ranjit × *O. rufipogon*, and Badshabhog × *O. rufipogon*] in this present investigation. Breeding lines were harvested and conserved in the Plant Genetics & Molecular Breeding Laboratory, Department of Botany; University of North Bengal, India.

### Hybridization and artificial pollination

Breeding lines were developed by artificial pollination and maintained for the present work. Hybridization was done in the year 2012 (October) based on standard protocol of artificial pollination^42–44^ for the development of breeding lines of different combinations. In brief- spikelet was emasculated by cutting at about one-third of the floret in a slanting position to expose the anthers and remove the anthers with the help of forceps. Emasculation was performed at 7 a.m. to 8 a.m. in the morning and covered with butter paper bag and tagged properly. Next day paper bag was removed and pollination was done (in between 11 a.m. to 3 p.m.) using a bunch of 200 male panicles with mature extrusion anthers which was collected from the field in the previous day and kept in water bucket. After pollination with this bunch of panicles to the emasculated female flowers were bagged again with the paper bag and tagged properly. After 25 days of pollination F1 seeds were collected from the parent plant (October 2012) and then subsequently maintained the lines for the development of F5 populations.

### Evaluation of agronomic traits

Morpho-agronomic traits were recorded according to DUS test protocol (PPV&FR Acts 2001, Govt. of India) for characteristics evaluation of the rice lines. The 21 days old seedlings of each line were transplanted with spacing 20 cm × 20 cm in randomized complete block design (RCBD) with 3 replications (Fig. 2). Observations like plant height, flag leaf length, flag leaf width, panicle length, grain per panicle, grain length, grain breadth, 1000 grain weight, maturity time (days), active tillering number, awn length and aroma were recorded.

### Scanning electron microscopy (SEM)

Mature grains were manually de-husked to take out the caryopsis. About ¼ th portion of both ends of the caryopses were removed and then fractured by applying slight pressure on the middle of the caryopsis with a razor blade for making solid round ring to get whole inner surface (*in situ* condition) of the grain. Caryopsis with the fractured surface (solid round ring) facing upwards were mounted on a specimen stub and coated with thin film of gold by means of a sputter coater (Jeol Model Smart Coater PF 18001006–2) about 2 minutes at high vacuum evaporator condition. Then ultra-structure was viewed with a scanning electron microscope (SEM) (Jeol Model JSM-IT100, Japan) at various magnification with an accelerating voltage of 10 kV^30–32^. The selected regions were then captured for further characterization of the morphological and histological properties using software InTouchScope of microscope control (Jeol). Total seventeen (17) rice caryopses of different breeding lines were used to reveal their morphological and histological characteristics at the *in situ* position of the microstructural components through scanning electron microscope (SEM) before and after cooking (retrograded).

### Cooking and retrogradation

The optimum cooking time was determined according to standard protocol^50^. Unpolished rice grain (caryopsis) in boiling water were taken out at a specific time interval during cooking and pressed between two glass slides until no opaque core or uncooked region was left. Then the cooked grain caryopsis was kept on glass slide for naturally retrogradation for 24 hours before SEM observation.

### Physicochemical properties and sensory based aroma test

Alkali spreading value (ASV) (in a scale of 1 to 7) was measured according to the standard method^51^. A low ASV corresponds to a high gelatinization temperature (GT), conversely, a high ASV indicates a low GT. Sensory based aroma (in a scale of 0 to 3) was evaluated using standard procedure^52^.

### Statistical data analysis and heritability measurement

The data were recorded in MS excel for the kharif crop 2016, 2017 and 2018. The data recorded for all the agronomic characters were subjected to ANOVA and then calculation for GCV (genotypic coefficient of variation), PCV (phenotypic coefficient of variation), heritability and genetic advance (GA) and genetic advance as percent of mean (GAM) analysis. Statistical analysis was done by using SPSS v.16.0. Genotypic and phenotypic variance was calculated using the formula of Lush^45^, and GCV, PCV were calculated by the formula of Burton^46^ and heritability as suggested^47,48^ and genetic advance (GA) computation based on the formula of Johnson *et al*.^49^.

## Supplementary information

Supplementary information.
