# Supplementary material for: Assessment of Genetic Heritability in Rice Breeding Lines Based on Morphological Traits and Caryopsis Ultrastructure
Source: Sci Rep. 2020 May 8;10:7830. doi: 10.1038/s41598-020-63976-8 (PMC7210993; doi:10.1038/s41598-020-63976-8)
Supplement: Supplementary file 1 — Supplementary information. [file 41598_2020_63976_MOESM1_ESM.doc]

## Supplementary information files

**Assessment of Genetic Heritability in Rice Breeding Lines Based on Morphological Traits and Caryopsis Ultrastructure**

Subhas Chandra Roy*and Pankaj Shil

Plant Genetics & Molecular Breeding Laboratory, Department of Botany, University of North Bengal, PO-NBU, Siliguri-734013, WB, India.

## Author information

### Affiliations

### Subhas Chandra Roy, Department of Botany, University of North Bengal, PO-NBU, Siliguri-734013, West Bengal, India.

### Pankaj Shil, C/o Prof. (Dr.) Subhas Chandra Roy. Department of Botany, University of North Bengal, PO-NBU, Siliguri-734013, West Bengal, India.

**Supplementary figures**


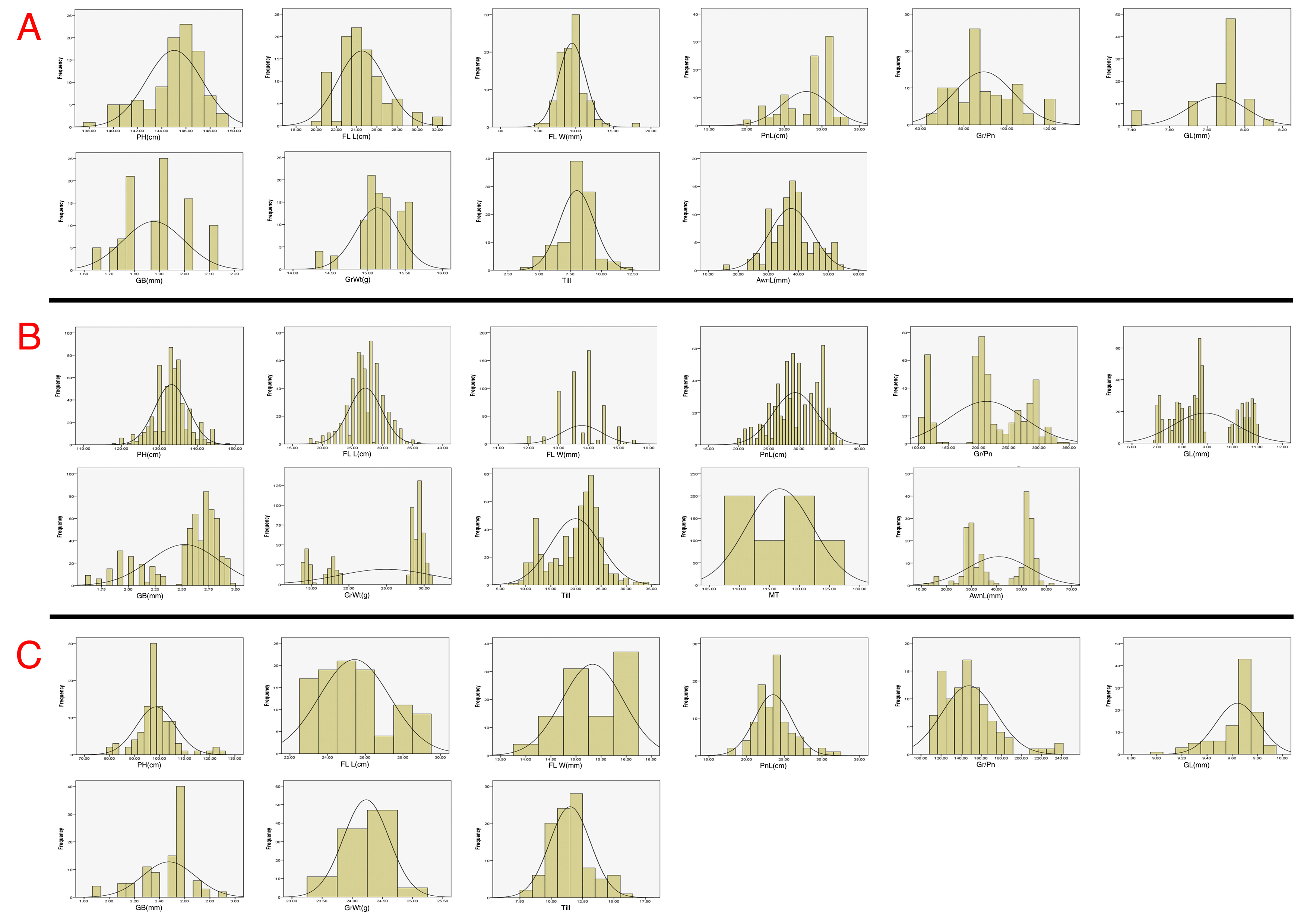


Supplementary Figure S1 Phenotypic distributions of the agronomic traits studied in this work. Histograms show data of the rice breeding lines at F_5:6_ generation of cross (Tulaipanji x IR64) considering the following agro-morphological traits- plant height (PH), flag leaf length (FLL), flag leaf width (FLW), panicle length (PnL), grain per panicle(Gr/Pn), grain length (GL), grain breadth (GB), 1000 grain weight (Gr/Wt), active tillering (Till), maturity time (MT), and awn length (AwnL). Panels for **A**: Tulaipanji, **B:** F5 progeny lines, **C:** IR64.


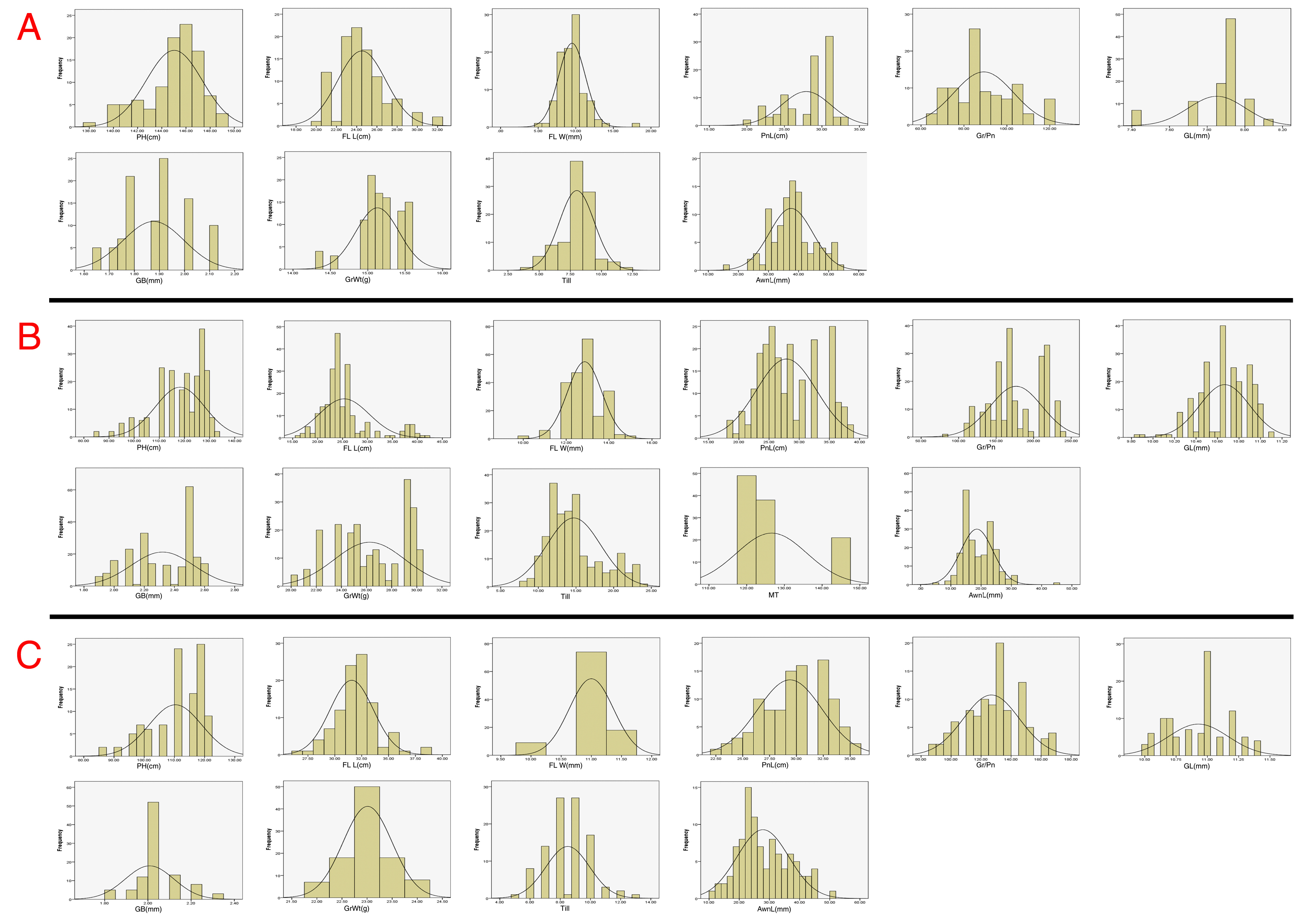


Supplementary Figure S2 Phenotypic distributions of the agronomic traits studied in this work. Histograms show data of the rice breeding lines at F_2:3_ generation of triparental cross (Tulaipanji x IR64 x PB1460) considering the following agro-morphological traits- plant height (PH), flag leaf length (FLL), flag leaf width (FLW), panicle length (PnL), grain per panicle(Gr/Pn), grain length (GL), grain breadth (GB), 1000 grain weight (Gr/Wt), active tillering (Till), maturity time (MT), and awn length (AwnL). Panels for **A**: Tulaipanji, **B:** F3 progeny lines, **C:** PB-1460.


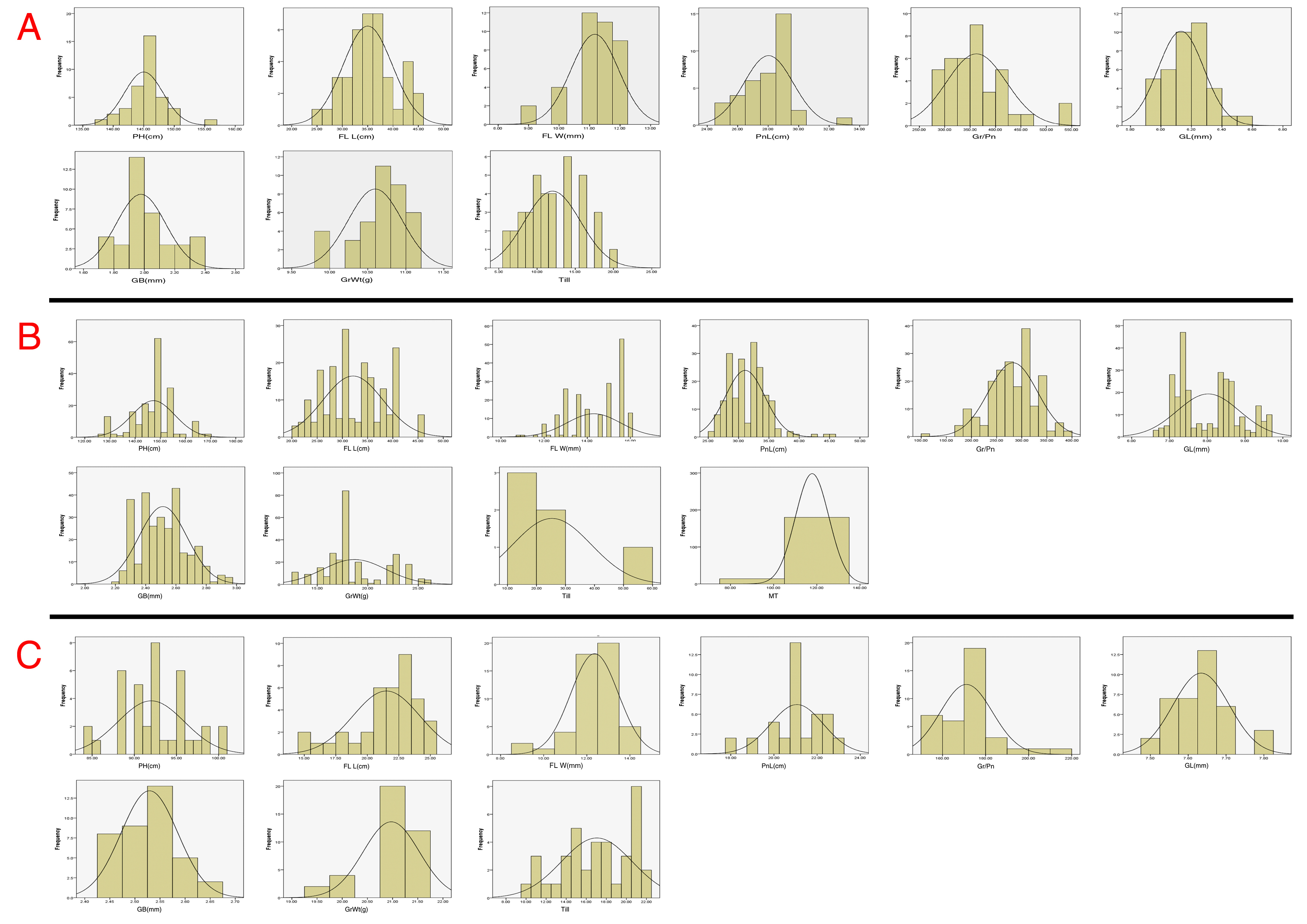


Supplementary Figure S3 Phenotypic distributions of the agronomic traits studied in this work. Histograms show data of the rice breeding lines at F_2:3_ generation of cross (Badshabhog x Swarna Sub1) considering the following agro-morphological traits- plant height (PH), flag leaf length (FLL), flag leaf width (FLW), panicle length (PnL), grain per panicle(Gr/Pn), grain length (GL), grain breadth (GB), 1000 grain weight (Gr/Wt), active tillering (Till), maturity time (MT), and awn length (AwnL). Panels for **A:** Badshabhog, **B:** F3 progeny lines, **C:** Swarna Sub1.


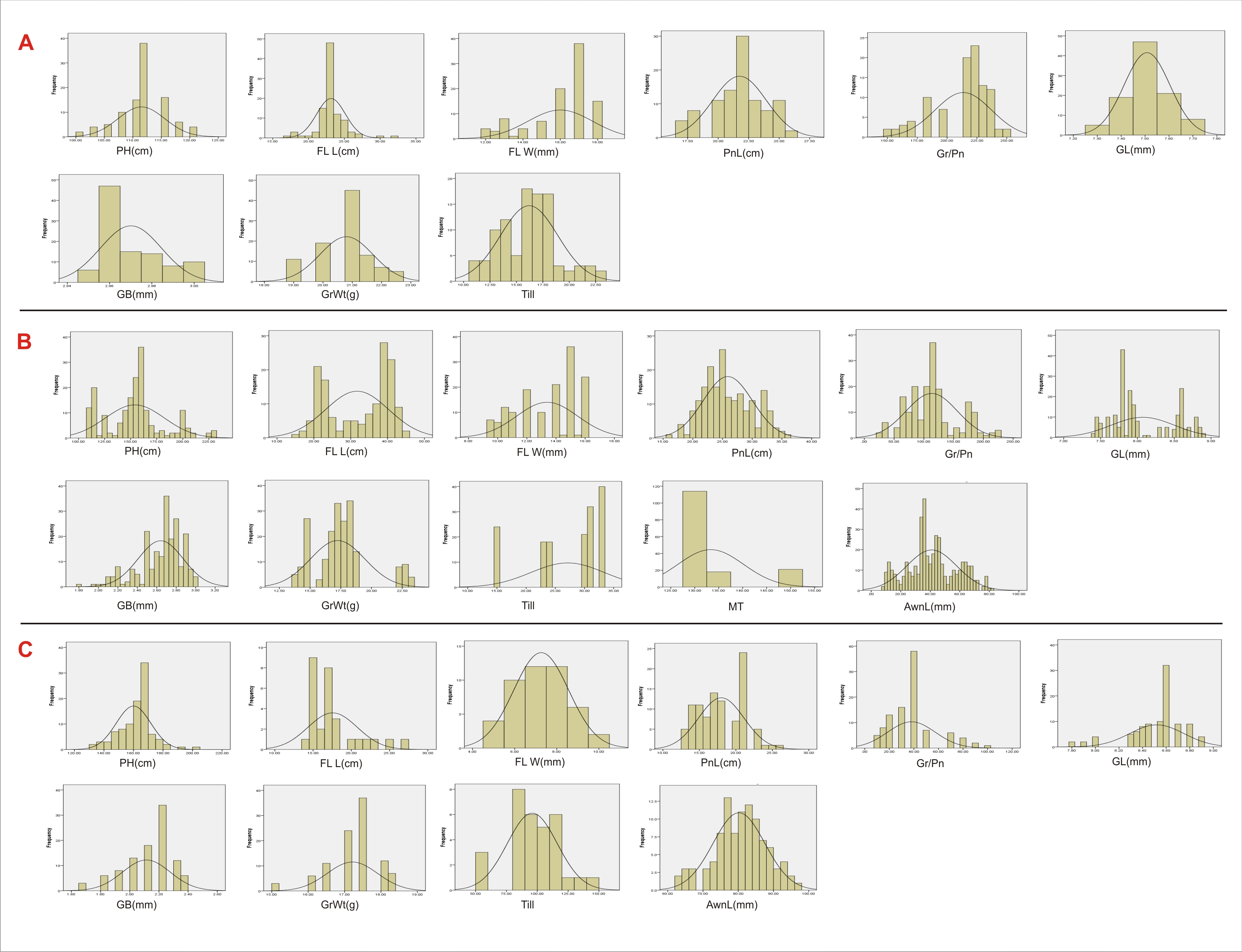


Supplementary Figure S4 Phenotypic distributions of the agronomic traits studied in this work. Histograms show data of the interspecific rice breeding lines at F_2:3_ generation of cross (Ranjit x *O. rufipogon*) considering the following agro-morphological traits- plant height (PH), flag leaf length (FLL), flag leaf width (FLW), panicle length (PnL), grain per panicle(Gr/Pn), grain length (GL), grain breadth (GB), 1000 grain weight (Gr/Wt), active tillering (Till), maturity time (MT), and awn length (AwnL). Panels for **A:** Ranjit, **B:** F3 progeny lines, **C:** *Oryza rufipogon* wild rice.


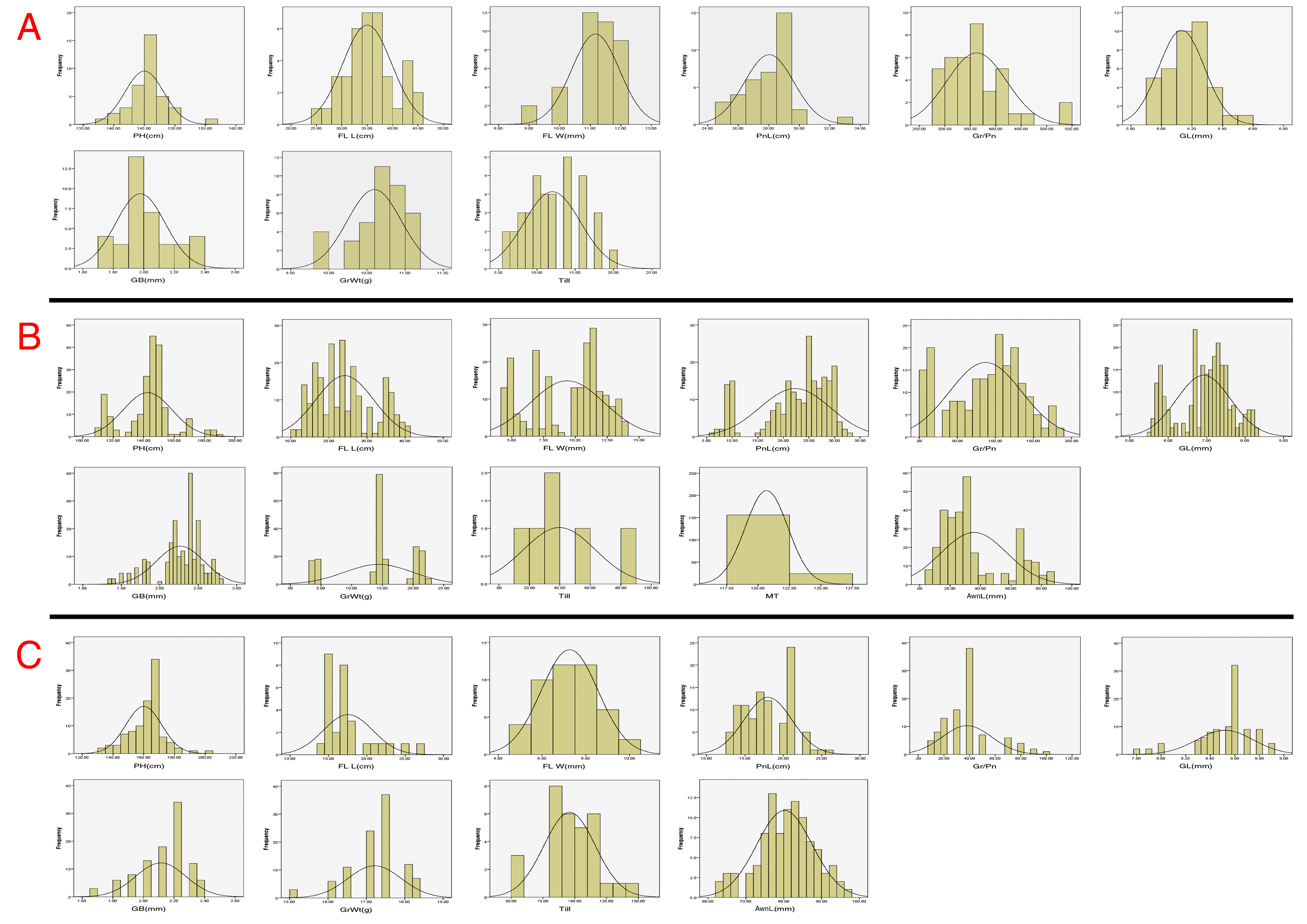


Supplementary Figure S5 Phenotypic distributions of the agronomic traits studied in this work. Histograms show data of the interspecific rice breeding lines at F_2:3_ generation of cross (Badshabhog x *O. rufipogon*) considering the following agro-morphological traits- plant height (PH), flag leaf length (FLL), flag leaf width (FLW), panicle length (PnL), grain per panicle(Gr/Pn), grain length (GL), grain breadth (GB), 1000 grain weight (Gr/Wt), active tillering (Till), maturity time (MT), and awn length (AwnL). Panels for **A:** Badshabhog, **B:** F3 progeny lines, **C:** *Oryza rufipogon* wild rice.

Supplementary Table S1. Statistical analysis of the morphological traits based on DUS test protocol of the rice breeding lines -F6L1ALMDG, F6L2AL, F6L3MDG, F6L4Aw, F6L5BrAw, F6L6BLK, of cross (Tulaipanji x IR64) at F_5:6_ generation. Plant height (PH), flag leaf length (FLL), flag leaf width (FLW), panicle length (PnL), grain per panicle(Gr/Pn), grain length (GL), grain breadth (GB), 1000 grain weight (Gr/Wt), active tillering (Till), maturity time (MT), and awn length (AwnL).

|  | **PH** | **FLL** | **FLW** | **PnL** | **Gr/Pn** | **GL** | **GB** | **GrWt** | **Till** | **MT** | **AwnL** | **Aroma** |
| --- | --- | --- | --- | --- | --- | --- | --- | --- | --- | --- | --- | --- |
| **Tulaipanji** | 145.00±0.30 | 24.50±0.42 | 9.50±0.13 | 27.9±0.32 | 95.07±1.39 | 7.85±0.02 | 1.87±0.01 | 15.12±0.02 | 8.02±0.70 | 150.00±0.00 | 37.5±0.02 | present |
| **F6 L1 AL MDG** | 133.50±0.26 | 27.60±0.14 | 13.83±0.04 | 33.16±0.16 | 202.0±0.61 | 8.57±0.01 | 2.76±0.009 | 29.91±0.05 | 21.00±0.07 | 115.00±0.02 | Nil | present |
| **F6 L2 AL** | 132.00±0.24 | 26.00±0.39 | 13.44±0.11 | 28.10±0.23 | 220.5±2.46 | 10.28±0.02 | 2.50±0.02 | 29.37±0.024 | 18.00±0.09 | 110.00±0.05 | Nil | present |
| **F6 L3 MDG** | 133.50±0.35 | 26.50±0.31 | 13.50±0.05 | 33.28±0.19 | 282.5±3.05 | 8.65±0.01 | 2.73±0.008 | 29.5±0.05 | 18.50±0.11 | 110.00±0.011 | Nil | present |
| **F6 L4 Aw** | 138.50±0.66 | 28.50±0.26 | 14.10±0.06 | 29.28±0.24 | 205.0±0.903 | 10.78±0.01 | 2.67±0.010 | 28.37±0.19 | 21.55±0.05 | 120.00±0.023 | 29.28±0.01 | present |
| **F6 L5 Br Aw** | 132.00±0.27 | 26.50±0.15 | 13.50±0.04 | 25.94±0.27 | 115.0±0.66 | 7.92±0.02 | 1.90±0.01 | 14.50±0.03 | 18.00±0.37 | 130.00±0.08 | 52.6±0.01 | present |
| **F6 L6 BLK** | 135.70±0.60 | 28.00±0.24 | 14.33±0.06 | 26.25±0.19 | 254.00±3.42 | 7.25±0.02 | 2.55±0.013 | 18.0±0.06 | 23.00±0.41 | 125.00±0.04 | Nil | Absent |
| **IR64** | 98.47±0.77 | 25.42±0.46 | 15.33±0.13 | 23.50±0.24 | 147.85±2.69 | 9.64±0.02 | 2.47±0.02 | 24.23±0.01 | 11.50±0.28 | 120.00±0.09 | Nil | Absent |

Mean of 5 plants, ± standard error

Supplementary Table S2. Statistical analysis of the morphological traits based on DUS test protocol of the rice breeding lines F1 line, F3i line, F3ii line of triparental cross (Tulaipanji x IR64 x PB1460) at F _2:3_ generation. Plant height (PH), flag leaf length (FLL), flag leaf width (FLW), panicle length (PnL), grain per panicle(Gr/Pn), grain length (GL), grain breadth (GB), 1000 grain weight (Gr/Wt), active tillering (Till), maturity time (MT), and awn length (AwnL).

|  | **PH** | **FLL** | **FLW** | **PnL** | **Gr/Pn** | **GL** | **GB** | **GrWt** | **Till** | **MT** | **AwnL** | **Aroma** |
| --- | --- | --- | --- | --- | --- | --- | --- | --- | --- | --- | --- | --- |
| **Tulaipanji** | 145.00±0.30 | 24.5±0.42 | 9.50±0.13 | 27.9±0.32 | 95.07±1.39 | 7.85±0.02 | 1.87±0.01 | 15.12±0.02 | 8.02±0.023 | 150.00±0.00 | 37.5±0.02 | present |
| **IR64** | 98.47±0.77 | 25.42±0.46 | 15.33±0.13 | 23.50±0.24 | 147.85±2.69 | 9.64±0.02 | 2.47±0.02 | 24.23±0.01 | 11.50±0.28 | 120.00±0.00 | Nil | Absent |
| **F1 line** | 125.29±0.64 | 38.44±0.89 | 13.26±0.12 | 23.45±0.37 | 152.19±3.63 | 10.36±0.05 | 2.12±0.03 | 26.5±0.10 | 15.00±0.00 | 145.00±0.00 | Nil | present |
| **F3i line** | 124.5±0.43 | 23.28±0.50 | 13.33±0.10 | 24.55±0.25 | 155.00±2.44 | 10.73±0.02 | 2.51±0.004 | 28.87±0.10 | 14.5±0.21 | 130.00±0.00 | 14.5±0.01 | present |
| **F3ii line** | 110.50±0.43 | 24.62±0.50 | 12.35±0.107 | 32.14±0.25 | 203.00±2.44 | 10.65±0.02 | 2.17±0.0049 | 23.58±0.215 | 15.33±0.00 | 120.00±0.00 | 22.66±0.18 | present |
| **PB1460** | 110.33±0.88 | 31.57±0.68 | 11.00±0.03 | 29.40±0.30 | 126.85±1.88 | 10.92±0.01 | 2.00±0.01 | 23.00±0.05 | 8.50±0.29 | 130.00±0.00 | 27.85±0.09 | present |

Mean of 5 plants, ± standard error

Supplementary Table S3. Statistical analysis of the morphological traits based on DUS test protocol of the rice breeding lines-BSF1A, BSF3A L1, BSF3A L2, BSF3A L3, BSF3A L4, BSF3A L5 of cross (Badshabhog x Swarna Sub1) at F_2:3_ generation. Plant height (PH), flag leaf length (FLL), flag leaf width (FLW), panicle length (PnL), grain per panicle(Gr/Pn), grain length (GL), grain breadth (GB), 1000 grain weight (Gr/Wt), active tillering (Till), maturity time (MT), and awn length (AwnL).

|  | **PH** | **FLL** | **FLW** | **PnL** | **Gr/Pn** | **GL** | **GB** | **GrWt** | **Till** | **MT** | **AwnL** | **Aroma** |
| --- | --- | --- | --- | --- | --- | --- | --- | --- | --- | --- | --- | --- |
| Badshabhog | 145.00±0.51 | 35.00±0.78 | 11.19±0.12 | 28.00±0.26 | 362.00±9.58 | 6.13±0.02 | 1.98±0.02 | 10.6±0.05 | 12.00±0.59 | 145±0.00 | Nil | present |
| BSF1A | 148.00±0.30 | 34.00±0.63 | 15.50±0.03 | 31.00±0.23 | 294.00±3.60 | 7.20±0.02 | 2.58±0.009 | 17.20±0.05 | 51.00±0.00 | 120.00±0.00 | Nil | Absent |
| BSF3A L1 | 163.74±0.79 | 34.54±0.56 | 12.22±0.17 | 35.72±1.28 | 336.33±15.91 | 9.4±0.03 | 2.75±0.02 | 24.00±0.15 | 16.00±0.00 | 90.00±0.00 | Nil | present |
| BSF3A L2 | 142.05±0.99 | 30.52±0.35 | 13.50±0.15 | 34.31±0.28 | 288.52±9.54 | 8.61±0.03 | 2.48±0.009 | 22.31±0.13 | 18.00±0.00 | 120.00±0.00 | Nil | present |
| BSF3A L3 | 142.28±0.78 | 28.78±0.52 | 13.57±0.11 | 30.00±0.54 | 264.78±10.33 | 7.50±0.03 | 2.30±0.006 | 14.25±0.15 | 16.00±0.00 | 120.00±0.00 | Nil | present |
| BSF3A L4 | 130.00±0.65 | 33.67±0.81 | 13.82±0.10 | 29.7±0.47 | 243.5±5.89 | 8.52±0.037 | 2.44±0.015 | 18.39±0.12 | 22.00±0.00 | 120.00±0.00 | Nil | present |
| BSF3A L5 | 153.85±0.40 | 24.33±0.36 | 12.87±0.08 | 27.56±0.22 | 241.7±7.76 | 8.35±0.021 | 2.37±0.01 | 18.19±0.11 | 29.00±0.00 | 120.00±0.00 | Nil | present |
| Swarna Sub 1 | 92.00±0.64 | 21.50±00.43 | 12.36±0.04 | 21.00±0.19 | 171.00±1.96 | 7.63±0.01 | 2.53±0.009 | 20.97±0.09 | 17.00±0.57 | 125.00±0.00 | Nil | Absent |

Mean of 5 plants, ± standard error

Supplementary Table S4. Statistical analysis of the morphological traits based on the DUS test protocol of the rice breeding lines –RWF1, RWF2 L1, RWF2 L2, RWF2 L3, RWF2 L4, RWF2 L5 at F_2:3_ generation (Ranjit x *O. rufipogon*). Plant height (PH), flag leaf length (FLL), flag leaf width (FLW), panicle length (PnL), grain per panicle(Gr/Pn), grain length (GL), grain breadth (GB), 1000 grain weight (Gr/Wt), active tillering (Till), maturity time (MT), and awn length (AwnL).

|  | **PH** | **FLL** | **FLW** | **PnL** | **Gr/Pn** | **GL** | **GB** | **GrWt** | **Till** | **MT** | **AwnL** | **Aroma** |
| --- | --- | --- | --- | --- | --- | --- | --- | --- | --- | --- | --- | --- |
| Ranjit | 111.5±0.41 | 23.14±0.77 | 16±0.42 | 21.75±0.21 | 213.57±2.37 | 7.5±0.02 | 2.97±0.008 | 20.8±0.090 | 16.2±0.78 | 125±0.00 | NIL | Absent |
| RWF1 | 132.33±1.98 | 21.66±0.27 | 10.21±0.11 | 28.85±0.26 | 68.59±1.36 | 7.86±0.05 | 2.14±0.03 | 22.43±0.08 | 30.00±0.00 | 150.00±0.00 | 71.0±1.01 | Absent |
| RWF2 L1 | 113.12±0.31 | 21.16±0.74 | 11.4±0.18 | 22.25±0.25 | 78.00±5.24 | 8.61±0.01 | 2.75±0.01 | 17.66±0.12 | 31.00±0.00 | 130.00±0.00 | 35.0±0.13 | Absent |
| RWF2 L2 | 158.5±0.75 | 30.7±0.76 | 13.87±0.19 | 24.83±0.47 | 98.6±4.92 | 7.75±0.02 | 2.70±0.01 | 17.33±0.13 | 15.00±0.00 | 130.00±0.00 | 52.5±0.31 | Absent |
| RWF2 L3 | 160.85±0.83 | 39.33±0.35 | 15.00±0.26 | 28.5±0.83 | 184.42±3.05 | 7.8±0.02 | 2.70±0.02 | 17.50±0.08 | 24.00±0.00 | 135.00±0.00 | 25±0.09 | Absent |
| RWF2 L4 | 201.83±2.42 | 37.5±0.65 | 14.2±0.15 | 28.77±0.74 | 125.00±5.02 | 8.68±0.02 | 2.86±0.01 | 14.33±0.06 | 23.00±0.00 | 130.00±0.00 | 45±0.02 | Absent |
| RWF2 L5 | 150.55±0.88 | 39.66±0.72 | 15.5±0.12 | 23.25±0.31 | 112.85±2.07 | 7.78±0.02 | 2.49±0.02 | 16.66±0.09 | 33.00±0.00 | 130.00±0.00 | Nil | Absent |
| *O. rufipogon* | 160.00±1.17 | 17.50±0.59 | 7.24±0.19 | 18.00±0.31 | 38.00±1.93 | 8.52±0.02 | 2.11±0.016 | 17.00±0.06 | 95.00±3.63 | 150.00±0.00 | 80.2±0.28 | Absent |

Mean of 5 plants, ± standard error

Supplementary Table S5. Statistical analysis of the morphological traits based on DUS test protocol of the rice breeding lines BWF1, BWF2 L1, BWF2 L2, BWF2 L3, BWF2 L4, BWF2 L5 at F _2:3_ generation (Badshabhog x *O. rufipogon*). Plant height (PH), flag leaf length (FLL), flag leaf width (FLW), panicle length (PnL), grain per panicle(Gr/Pn), grain length (GL), grain breadth (GB), 1000 grain weight (Gr/Wt), active tillering (Till), maturity time (MT), and awn length (AwnL).

|  | **PH** | **FLL** | **FLW** | **PnL** | **Gr/Pn** | **GL** | **GB** | **GrWt** | **Till** | **MT** | **AwnL** | **Aroma** |
| --- | --- | --- | --- | --- | --- | --- | --- | --- | --- | --- | --- | --- |
| **Badshabhog** | 145.00±0.51 | 35.00±0.78 | 11.19±0.12 | 28.00±0.26 | 362.00±9.58 | 6.13±0.02 | 1.98±0.02 | 10.6±0.05 | 12.00±0.59 | 145±0.00 | Nil | present |
| **BWF1** | 145.00±0.59 | 17.80±0.43 | 7.24±0.14 | 22.00±0.44 | 63.00±3.35 | 6.86±0.03 | 2.19±0.007 | 14.1±0.02 | 80.00±0.00 | 120.00±0.00 | 30.50±0.032 | Absent |
| **BWF2 L1** | 115.16±0.43 | 20.85±0.97 | 4.8±0.05 | 9.00±0.15 | 10.85±0.74 | 5.75±0.01 | 1.85±0.02 | 13.00±0.08 | 35.00±0.00 | 120.00±0.00 | 21.76±0.121 | Present |
| **BWF2 L2** | 147.12±0.62 | 22.00±0.56 | 12.85±0.15 | 26.9±0.55 | 119.15±3.59 | 7.93±0.04 | 2.38±0.02 | 21.2±0.09 | 340.00±0.00 | 120.00±0.00 | 66.71±0.110 | Absent |
| **BWF2 L3** | 142.64±0.93 | 33.85±0.72 | 11.5±0.09 | 24.93±0.40 | 105.83±2.95 | 7.33±0.02 | 2.42±0.007 | 14.5±0.06 | 52.00±0.00 | 120.00±0.00 | 16.33±0.201 | Absent |
| **BWF2 L4** | 148.70±0.62 | 22.3±0.949 | 10.25±0.09 | 27.05±0.09 | 159.71±7.61 | 6.42±0.05 | 2.45±0.03 | 14.00±0.16 | 13.00±0.00 | 120.00±0.00 | Nil | Absent |
| **BWF2 L5** | 168.55±2.62 | 28.82±1.07 | 11.31±0.06 | 29.18±0.60 | 120.00±5.14 | 7.03±0.04 | 2.67±0.01 | 20.00±0.08 | 24.00±0.00 | 125.00±0.00 | Nil | Absent |
| ***O. rufipogon*** | 160.00±1.17 | 17.5±0.59 | 7.24±0.19 | 18.00±0.31 | 38.00±1.93 | 8.52±0.02 | 2.11±0.016 | 17.00±0.06 | 95.00±3.63 | 150.00±0.00 | 80.2±0.037 | Absent |

Mean of 5 plants, ± standard error

Supplementary Table S6 Correlation among the parameters of different agro-morphological traits of the rice breeding lines of cross (Tulaipanji x IR64) for F_5:6_ populations. Plant height (PH), flag leaf length (FLL), flag leaf width (FLW), panicle length (PnL), grain per panicle(Gr/Pn), grain length (GL), grain breadth (GB), 1000 grain weight (Gr/Wt), active tillering (Till), heading date (HD), and maturity time (MT).

|  | **PH** | **FLL** | **FLW** | **PnL** | **Gr/Pn** | **GL** | **GB** | **GrWt** | **Till** | **HD** | **MT** |
| --- | --- | --- | --- | --- | --- | --- | --- | --- | --- | --- | --- |
| **PH** | **1** | **.493^**^** | **.458^**^** | **.606^**^** | **.315^**^** | **.128^**^** | **.409^**^** | **.139^**^** | **.493^**^** | **-0.05** | **.130^**^** |
| **FLL** |  | **1** | **.724^**^** | **.350^**^** | **0.067** | **.240^**^** | **.328^**^** | **0.072** | **.371^**^** | **.122^**^** | **.101^*^** |
| **FLW** |  |  | **1** | **.391^**^** | **.231^**^** | **.469^**^** | **.431^**^** | **.276^**^** | **.497^**^** | **0.017** | **.326^**^** |
| **PnL** |  |  |  | **1** | **.690^**^** | **-.101^**^** | **.783^**^** | **.557^**^** | **.291^**^** | **-.539^**^** | **.473^**^** |
| **Gr/Pn** |  |  |  |  | **1** | **.229^**^** | **.813^**^** | **.812^**^** | **0.037** | **-.756^**^** | **.736^**^** |
| **GL** |  |  |  |  |  | **1** | **.411^**^** | **.483^**^** | **.424^**^** | **0.056** | **.607^**^** |
| **GB** |  |  |  |  |  |  | **1** | **.897^**^** | **.436^**^** | **-.607^**^** | **.876^**^** |
| **GrWt** |  |  |  |  |  |  |  | **1** | **.296^**^** | **-.746^**^** | **.969^**^** |
| **Till** |  |  |  |  |  |  |  |  | **1** | **.202^**^** | **.441^**^** |
| **HD** |  |  |  |  |  |  |  |  |  | **1** | **-.591^**^** |
| **MT** |  |  |  |  |  |  |  |  |  |  | **1** |

Significant at p<0.05 (*) and p<0.01 (**).

Supplementary Table S7 Correlation among the parameters of different agro-morphological traits of the rice breeding lines of triparental cross (Tulaipanji x IR64 x PB1460) for F_2:3_ populations. Plant height (PH), flag leaf length (FLL), flag leaf width (FLW), panicle length (PnL), grain per panicle(Gr/Pn), grain length (GL), grain breadth (GB), 1000 grain weight (Gr/Wt), active tillering (Till), heading date (HD), and maturity time (MT).

|  | **PH** | **FLL** | **FLW** | **PnL** | **Gr/Pn** | **GL** | **GB** | **GrWt** | **Till** | **HD** | **MT** |
| --- | --- | --- | --- | --- | --- | --- | --- | --- | --- | --- | --- |
| **PH** | **1** | **.640^**^** | **.820^**^** | **-0.097** | **-0.017** | **.764^**^** | **.949^**^** | **.928^**^** | **-.502^**^** | **.818^**^** | **.818^**^** |
| **FLL** |  | **1** | **.749^**^** | **.630^**^** | **.595^**^** | **.931^**^** | **.340^**^** | **.372^**^** | **.249^*^** | **0.208** | **0.208** |
| **FLW** |  |  | **1** | **-.241^*^** | **-0.194** | **.617^**^** | **.754^**^** | **.817^**^** | **-.235^*^** | **.667^**^** | **.667^**^** |
| **PnL** |  |  |  | **1** | **.958^**^** | **.466^**^** | **-.316^**^** | **-.418^**^** | **.820^**^** | **-.830^**^** | **-.830^**^** |
| **Gr/Pn** |  |  |  |  | **1** | **.523^**^** | **-.254^**^** | **-.336^**^** | **.750^**^** | **-.773^**^** | **-.773^**^** |
| **GL** |  |  |  |  |  | **1** | **.613^**^** | **.567^**^** | **.263^*^** | **0.176** | **0.176** |
| **GB** |  |  |  |  |  |  | **1** | **.960^**^** | **-.666^**^** | **.935^**^** | **.935^**^** |
| **GrWt** |  |  |  |  |  |  |  | **1** | **-.579^**^** | **.931^**^** | **.931^**^** |
| **Till** |  |  |  |  |  |  |  |  | **1** | **-.778^**^** | **-.778^**^** |
| **HD** |  |  |  |  |  |  |  |  |  | **1** | **1.000^**^** |
| **MT** |  |  |  |  |  |  |  |  |  |  | **1** |

Significant at p<0.05 (*) and p<0.01 (**).

Supplementary Table S8 Correlation among the parameters of different agro-morphological traits of the rice breeding lines of cross (Badshabhog x Swarna Sub1) in F_2:3_ populations. Plant height (PH), flag leaf length (FLL), flag leaf width (FLW), panicle length (PnL), grain per panicle(Gr/Pn), grain length (GL), grain breadth (GB), 1000 grain weight (Gr/Wt), active tillering (Till), heading date (HD), maturity time (MT).

|  | **PH** | **FLL** | **FLW** | **PnL** | **Gr/Pn** | **GL** | **GB** | **GrWt** | **Till** | **HD** | **MT** |
| --- | --- | --- | --- | --- | --- | --- | --- | --- | --- | --- | --- |
| **PH** | **1** | **.599^**^** | **.279^**^** | **.242^**^** | **.703^**^** | **0.119** | **-.291^**^** | **-.304^**^** | **.177^*^** | **-.226^**^** | **-.165^*^** |
| **FLL** |  | **1** | **0.127** | **.443^**^** | **.627^**^** | **.291^**^** | **0.038** | **-0.026** | **-0.032** | **-.349^**^** | **-.303^**^** |
| **FLW** |  |  | **1** | **-.764^**^** | **.715^**^** | **-.809^**^** | **-.743^**^** | **-.810^**^** | **-.476^**^** | **.704^**^** | **.769^**^** |
| **PnL** |  |  |  | **1** | **-0.149** | **.914^**^** | **.706^**^** | **.699^**^** | **.482^**^** | **-.834^**^** | **-.862^**^** |
| **Gr/Pn** |  |  |  |  | **1** | **-.272^**^** | **-.374^**^** | **-.483^**^** | **-.260^**^** | **0.144** | **.221^**^** |
| **GL** |  |  |  |  |  | **1** | **.820^**^** | **.851^**^** | **.609^**^** | **-.870^**^** | **-.899^**^** |
| **GB** |  |  |  |  |  |  | **1** | **.908^**^** | **.456^**^** | **-.721^**^** | **-.764^**^** |
| **GrWt** |  |  |  |  |  |  |  | **1** | **.383^**^** | **-.726^**^** | **-.767^**^** |
| **Till** |  |  |  |  |  |  |  |  | **1** | **-.318^**^** | **-.366^**^** |
| **HD** |  |  |  |  |  |  |  |  |  | **1** | **.995^**^** |
| **MT** |  |  |  |  |  |  |  |  |  |  | **1** |

Significant at p<0.05 (*) and p<0.01 (**).

Supplementary Table S9 Correlation among the parameters of different agro-morphological traits of the interspecific rice breeding lines (Ranjit x *O. rufipogon*) for F_2:3_ populations. Plant height (PH), flag leaf length (FLL), flag leaf width (FLW), panicle length (PnL), grain per panicle(Gr/Pn), grain length (GL), grain breadth (GB), 1000 grain weight (Gr/Wt), active tillering (Till), heading date (HD), and maturity time (MT).

|  | **PH** | **FLL** | **FLW** | **PnL** | **Gr/Pn** | **GL** | **GB** | **GrWt** | **Till** | **HD** | **MT** |
| --- | --- | --- | --- | --- | --- | --- | --- | --- | --- | --- | --- |
| **PH** | **1** | **.666^**^** | **.562^**^** | **.647^**^** | **.476^**^** | **0.111** | **.392^**^** | **0.084** | **-.458^**^** | **-.632^**^** | **0.147** |
| **FLL** |  | **1** | **.904^**^** | **.544^**^** | **.746^**^** | **-.210^*^** | **-0.054** | **-0.052** | **0.021** | **-.353^**^** | **.377^**^** |
| **FLW** |  |  | **1** | **.564^**^** | **.735^**^** | **-.264^**^** | **-0.079** | **-0.132** | **0.027** | **-.388^**^** | **.286^**^** |
| **PnL** |  |  |  | **1** | **.772^**^** | **.228^**^** | **.632^**^** | **.595^**^** | **-.210^*^** | **-0.059** | **.284^**^** |
| **Gr/Pn** |  |  |  |  | **1** | **-0.14** | **.287^**^** | **.549^**^** | **-0.103** | **0.131** | **.730^**^** |
| **GL** |  |  |  |  |  | **1** | **.714^**^** | **.352^**^** | **.320^**^** | **.351^**^** | **-.359^**^** |
| **GB** |  |  |  |  |  |  | **1** | **.585^**^** | **-.293^**^** | **.264^**^** | **-0.08** |
| **GrWt** |  |  |  |  |  |  |  | **1** | **0.023** | **.650^**^** | **.418^**^** |
| **Till** |  |  |  |  |  |  |  |  | **1** | **0.167** | **-.217^*^** |
| **HD** |  |  |  |  |  |  |  |  |  | **1** | **.516^**^** |
| **MT** |  |  |  |  |  |  |  |  |  |  | **1** |

Significant at p<0.05 (*) and p<0.01 (**).

Supplementary Table S10 Correlation among the parameters of different agro-morphological traits of the interspecific rice breeding lines (Badshabhog x *O. rufipogon*) for F_2:3_ populations. Plant height (PH), flag leaf length (FLL), flag leaf width (FLW), panicle length (PnL), grain per panicle(Gr/Pn), grain length (GL), grain breadth (GB), 1000 grain weight (Gr/Wt), active tillering (Till), heading date (HD), and maturity time (MT).

|  | **PH** | **FLL** | **FLW** | **PnL** | **Gr/Pn** | **GL** | **GB** | **GrWt** | **Till** | **HD** | **MT** |
| --- | --- | --- | --- | --- | --- | --- | --- | --- | --- | --- | --- |
| **PH** | **1** | **.476^**^** | **.780^**^** | **.902^**^** | **.861^**^** | **.662^**^** | **.917^**^** | **.823^**^** | **-.244^**^** | **.627^**^** | **.627^**^** |
| **FLL** |  | **1** | **.434^**^** | **.510^**^** | **.478^**^** | **.420^**^** | **.582^**^** | **.211^**^** | **.470^**^** | **0.125** | **0.125** |
| **FLW** |  |  | **1** | **.941^**^** | **.861^**^** | **.949^**^** | **.873^**^** | **.923^**^** | **0.124** | **0.136** | **0.136** |
| **PnL** |  |  |  | **1** | **.958^**^** | **.841^**^** | **.951^**^** | **.881^**^** | **-0.063** | **.306^**^** | **.306^**^** |
| **Gr/Pn** |  |  |  |  | **1** | **.724^**^** | **.898^**^** | **.781^**^** | **-0.155** | **.225^**^** | **.225^**^** |
| **GL** |  |  |  |  |  | **1** | **.749^**^** | **.881^**^** | **.248^**^** | **0.021** | **0.021** |
| **GB** |  |  |  |  |  |  | **1** | **.841^**^** | **-0.053** | **.454^**^** | **.454^**^** |
| **GrWt** |  |  |  |  |  |  |  | **1** | **-0.128** | **.358^**^** | **.358^**^** |
| **Till** |  |  |  |  |  |  |  |  | **1** | **-.457^**^** | **-.457^**^** |
| **HD** |  |  |  |  |  |  |  |  |  | **1** | **1.000^**^** |
| **MT** |  |  |  |  |  |  |  |  |  |  | **1** |

Significant at p<0.05 (*) and p<0.01 (**).
